# Phase-dependent word perception emerges from region-specific sensitivity to the statistics of language

**DOI:** 10.1073/pnas.2320489121

**Published:** 2024-05-28

**Authors:** Sanne Ten Oever, Lorenzo Titone, Noémie te Rietmolen, Andrea E. Martin

**Affiliations:** ^a^Language and Computation in Neural Systems group, Max Planck Institute for Psycholinguistics, Nijmegen XD 6525, The Netherlands; ^b^Language and Computation in Neural Systems group, Donders Centre for Cognitive Neuroimaging, Donders Institute for Brain, Cognition and Behaviour, Radboud University, Nijmegen EN 6525, The Netherlands; ^c^Department of Cognitive Neuroscience, Faculty of Psychology and Neuroscience, Maastricht University, EV 6229, The Netherlands; ^d^Research Group Language Cycles, Max Planck Institute for Human Cognitive and Brain Sciences, Leipzig D-04303, Germany

**Keywords:** speech, spoken word recognition, neural oscillations, phase, MEG

## Abstract

Neural oscillations reflect fluctuations in excitability, which biases the percept of ambiguous sensory input. Why this bias occurs is still not fully understood. We hypothesized that neural populations representing likely events are more sensitive, and thereby become active on earlier oscillatory phases, when the ensemble itself is less excitable. Perception of ambiguous input presented during less-excitable phases should therefore be biased toward frequent or predictable stimuli that have lower activation thresholds. Here, we show such a frequency bias in spoken word recognition using psychophysics, magnetoencephalography (MEG), and computational modelling. With MEG, we found a double dissociation, where the phase of oscillations in the superior temporal gyrus and medial temporal gyrus biased word-identification behavior based on phoneme and lexical frequencies, respectively. This finding was reproduced in a computational model. These results demonstrate that oscillations provide a temporal ordering of neural activity based on the sensitivity of separable neural populations.

## Significance

Previous work has suggested that the phase of ongoing neural oscillations is important for the separation of neural representations and has a direct influence on perceptual decisions during speech perception. It is unknown what the nature of this phase code is. Here, we show that phase coding is based on the probability of an interpreted speech event, here a linguistic unit, such that highly probable events appear to be coded at lower excitability phases; we additionally demonstrate that this phase code is region specific. These results suggest that oscillations separate linguistic neural representations based on the excitability of neuronal populations.

Oscillations, or population rhythmic activity, reflect the waxing and waning of neural excitability such that individual neurons modulated by an oscillation are primarily active on high excitable phases (1–3). Previous studies have directly linked this phase-dependent neural activity to behavioral performance showing that target detection (4–7) and accuracy in categorization tasks (8, 9) are modulated by oscillatory phase [note also that there have been a substantial amount of null findings regarding phase-dependent effects (10–12)]. Besides accuracy, a few studies have also shown that oscillatory phase can modulate the categorization of ambiguous stimuli by biasing participants’ percept to one or another category based on the phase of presentation (13–15). Improved behavioral performance has often been attributed to increased processing efficiency at oscillatory phases at which neural activity is increased (3, 16). However, phase-dependent categorization biases cannot be explained by overall increases in activity (or increased processing efficiency) on specific oscillatory phases because increases in overall activity should not bias processing to one specific perceptual interpretation. Thus, it is unclear what neural mechanism underlies phase-dependent categorization.

Even though oscillations modulate neural excitability, not all neurons influenced by an oscillation reach activation exactly at the same time or phase. In fact, the phase of firing of a neuron is determined by an interaction between excitability changes due to oscillations and the neural sensitivity of a neuron to incoming signals (17). Neurons that are sensitive to incoming signals will respond strongly and will therefore reach activation already at relatively low excitable oscillatory phases (1, 17). In contrast, neurons less sensitive to the input will reach activation only at later, more excitable, phases. In this way, different neurons are active at different phases, which has been proposed to serve as a mechanism to separate neural representations and their readouts (18). Neuronal sensitivity can be modulated by neuroplastic changes induced through associative and statistical learning (19). For example, neural populations representing more likely events in the world have higher sensitivity than populations representing less likely events (19, 20). If this is true, populations representing probable events should be active at earlier, less excitable, oscillatory phases compared to populations representing less likely events which in turn could lead to phase-dependent categorization (21).

Previously, we have shown that oscillatory phase in the theta frequency range can bias the categorization of an ambiguous syllable (14). In that study, we presented an ambiguous syllable that Dutch participants could interpret as /dα/ or /xα/ [notation according to the international phonetic alphabet (IPA)]. Originally, this phase-dependent categorization bias was attributed to an articulatory visual-to-auditory temporal difference between the two syllables (the visual-to-auditory articulatory delay of /dα/ is shorter than /xα/) (14, 22). However, in Dutch, /d/ also has a higher frequency than /x/ (23), that is, /d/ is more probable than /x/. It is therefore possible that the categorization effect in this study was instead (or additionally) caused by an interaction between ongoing oscillations and the sensitivity of neural populations for these consonants (21). If oscillations provide a temporal ordering based on neural sensitivity (17, 21), then one would expect phasic categorization effects to occur when there is a difference in event probability for two possible interpretations of an ambiguous stimulus. If this is so, we should view oscillations not merely as a gating operation opening and closing lines of neural communication (3, 16), but rather as a rich source of representational space (18, 24).

**Fig. 1.**
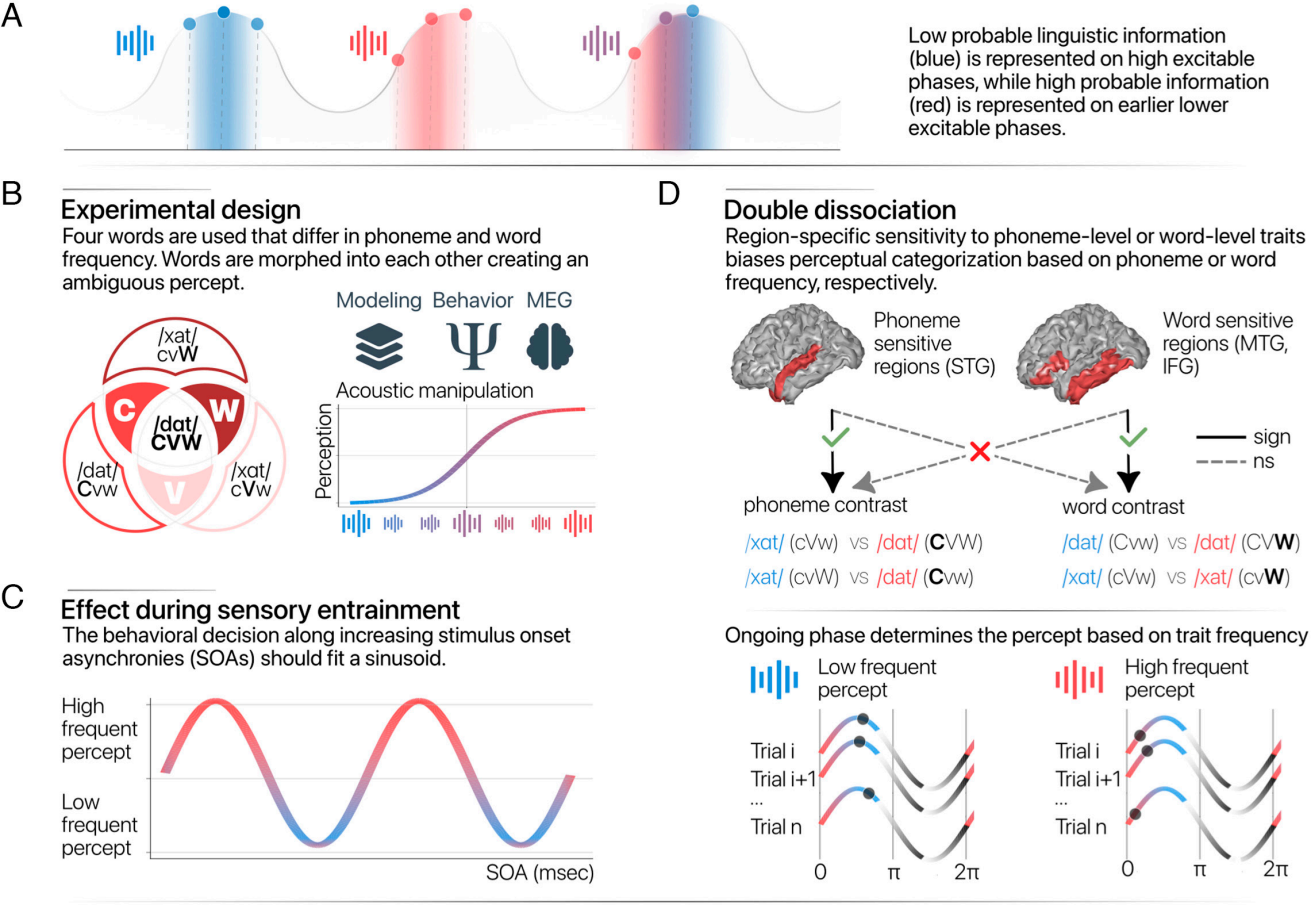
Overview of the current study. (*A*) It is hypothesized that low probable linguistic information is represented at high excitable phases, while high probable linguistic information is represented at low excitable phases. The perceived sound of ambiguous stimuli depends on the phase of presentation. (*B*) Four words are used that differ in consonant (C), vowel (V), and word (W) frequency. Words are morphed into each other creating an ambiguous percept. (*C*) Sensory entrainment locks neural rhythms to the rhythmic input and therefore the stimulus onset asynchrony (SOA) relative to the entrainment train should bias the percept to words containing low or high frequent linguistic information. (*D*) In MEG, a double dissociation is expected in which perceptual bias is governed by phoneme or word frequency for regions sensitive to phonemic or word features, respectively. Sign = significant; ns = not significant. STG = superior temporal gyrus; MTG = middle temporal gyrus; IFG = inferior frontal gyrus.

To investigate the relation between phase-dependent categorization and event probability, we presented participants with words that varied in consonant, vowel, and word frequency. These were the four Dutch words *dat*, *gat*, *daad*, and *gaat* (see Table 1 for translations, IPA notation, and event frequencies; also see *SI Appendix*, Fig. S1 for word-position specific event frequencies). In this way, we manipulated event probabilities at different levels of analysis based on the recurrence of phonemic and lexical elements in a language. By using psychophysics, MEG, and computational modeling, we could investigate the influence of event probability on behavioral and neural responses to ambiguous stimuli (Fig. 1). Psychophysics showed phase-dependent behavioral responses when one of the response options had low lexical and phonemic frequency and the other option had high lexical and phonemic frequency. With MEG, we could further disentangle lexical and phonemic frequency effects by investigating neural sources that are more sensitive to one of the features. Here, we again showed that phase biases categorization when words had different probabilities of occurrence. Moreover, we found a dissociation in which the phase of oscillations in the superior temporal gyrus (STG) and medial temporal gyrus (MTG) biased word-identification behavior based on phoneme and lexical frequencies, respectively. These outcomes were verified using computational modeling.

**Table 1.** Stimulus materials

Dutch word	*gat*	*dat*	*gaat*	*daad*
Translation	*hole*	*that*	*go*	*deed*
IPA	/xαt/	/dαt/	/xat/	/dat/
Consonant frequency	−2.53 (−)	−1.87 (+)	−2.53 (−)	−1.87 (+)
Vowel frequency	−1.96 (+)	−1.96 (+)	−2.29 (−)	−2.29 (−)
Word frequency	−10.47 (−)	−3.82 (+)	−6.41 (+)	−11.66 (−)
Frequency denotation	cVw	CVW	cvW	Cvw

Four different Dutch words used in the study. Frequency is indicated on a log scaled based on instance occurrence in the Corpus Gesproken Nederlands. Less negative numbers indicate a higher frequency. The frequency is indicated for each class as being high (+) or low(-) IPA = international phonetic alphabet. In the rest of the text, we will denote the frequency across the three traits [consonant (C), vowel (V), and word (W)] by indicating via lower- or upper-case letters whether the word has a low (lower-case) or high (upper-case) frequency for a specific trait (see denotation at the last row).

## Results

### Psychophysics Experiment.

**Fig. 2.**
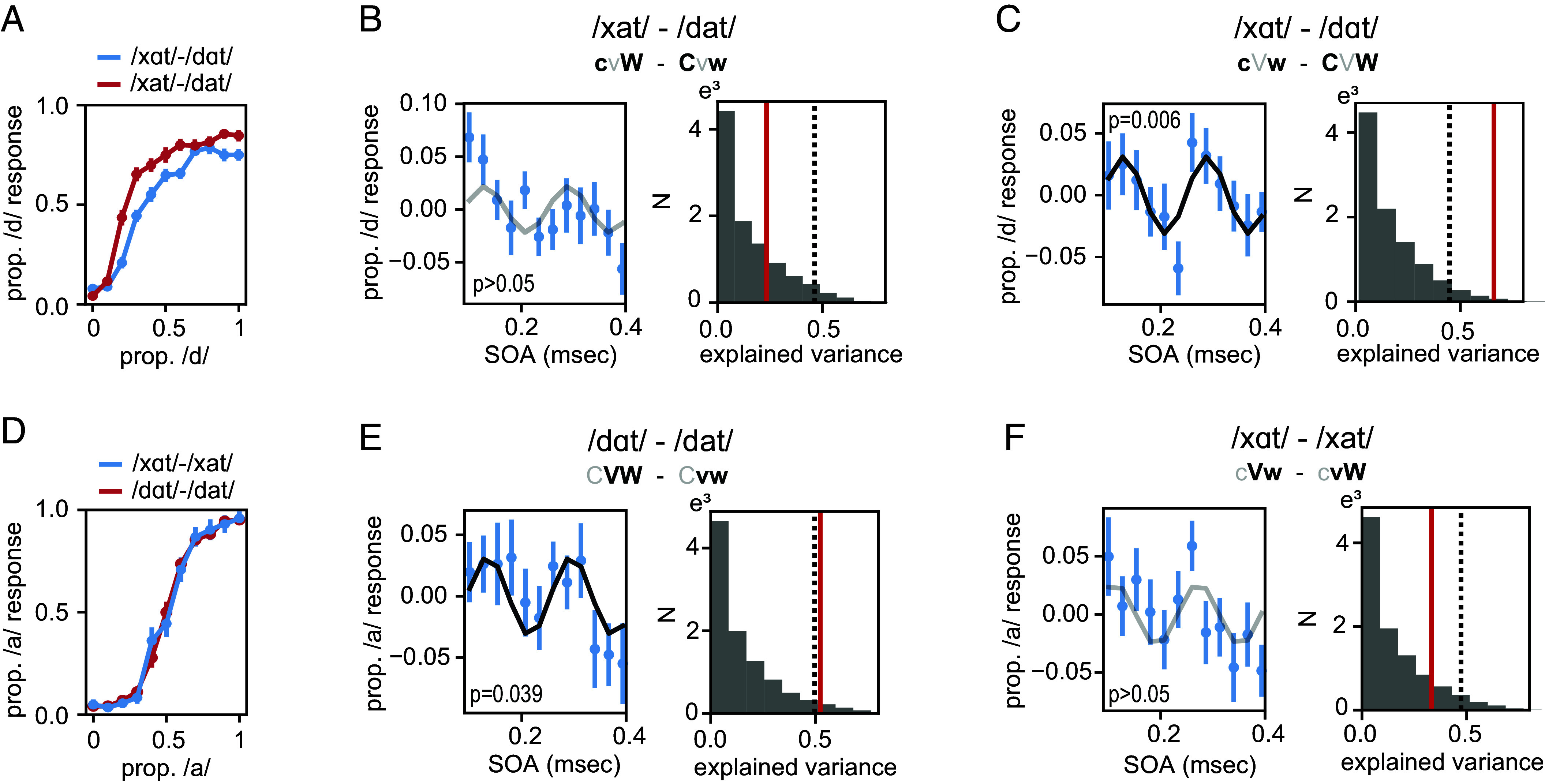
Behavioral results. (*A*) Psychometric curves for the consonant experiment (separate lines for the two vowel types). (*B*) Outcome of the main experiment for the /xat/-/dat/ spectrum. The *Left* panel shows the average demeaned time course across SOA. The *Right* panel shows a histogram of the null distribution together with the observed explained variance of the sinusoid fit (red line) and the 95th percentile (dotted line) of the null distribution. (*C*) Same as *B* for the /xαt/-/dαt/ spectrum. (*D*–*F*) Same as *A*–*C* for the vowel experiment. Error bars indicate the SEM. Black lines indicate the best fitted curve (gray if not significant).

We investigated whether combining oscillations with dissimilar “event” probabilities—here phoneme and lexical frequency—can lead to phase-dependent categorization. We presented an entrainment stream at 6.25 Hz after which an ambiguous word was presented at a variable stimulus onset asynchronies (SOAs). Assuming an entrained oscillation, these SOAs match to ongoing oscillatory phases (25). Ambiguous words were generated by creating 10 equally spaced morph levels along either the consonant (/x/-/d/) or the vowel dimension (/a/-/α/), resulting in four morphs: /xat/-/dat/, /xαt/-/dαt/, /dαt/-/dat/, and /xαt/-/xat/. During the first part of the experiment, we presented stimuli across all morphs to create an individual psychometric curve along the consonant (Fig. 2*A*) and vowel dimension (Fig. 2*D*). Only participants for which we could reliably extract an ambiguous stimulus via fitting a psychometric curve could participate in the main experiment (included participants were n = 18 and n = 12 for the consonant and vowel version, respectively). Participants gave informed consent online. The study was approved by the Ethics Board of the Social Sciences Faculty of Radboud University in Nijmegen. Participants received monetary reimbursement for their participation.

In the main experiment, participants listened to rhythmic broadband noise bursts presented at 6.25 Hz after which an ambiguous word was presented. The SOAs at which ambiguous words were presented ranged between 0.1 and 0.4 s in 12 equidistant steps (spanning exactly two cycles of 6.25 Hz). Participants had to indicate which word they heard. Under the null hypothesis, we would expect no 6.25 Hz modulation of the response patterns based on the SOA, while this modulation was expected under the alternative hypothesis. To test this, we fitted a sinusoid at 6.25 Hz to the demeaned data and compared this to a null distribution based on random permutations across the SOAs and repeating the fit (see *SI Appendix*, Fig. S2 for nondemeaned data). For the consonant experiment, we found that the 6.25 Hz sinusoid fitted to the data yielded a higher explained variance than expected by chance for the /xαt/-/dαt/ morph (*P* = 0.006, r^2^ = 0.661; Fig. 2*C*), but not for the /xat/-/dat/ morph (*P* = 0.258; Fig. 2*B*). For the vowel experiment, we could significantly fit a sinusoid for the /dαt/-/dat/ (*P* = 0.039, r^2^ = 0.523; Fig. 2*E*), but not the /xαt/-/xat/ morph (*P* = 0.136; Fig. 2*F*). In sum, we could only fit a significant curve for morphs in which both varied traits had opposing frequencies in the word pairs, that is for the /xαt/-/dαt/, cVw – CVW, morph and the /dαt/-/dat/, CVW – Cvw, morph.

### MEG Experiment.

In the psychophysics experiment, all responses are based on the integration of information across both the phonemic and lexical level of analyses as there is only one behavioral output. However, it would be expected that regions primarily sensitive to phonemic frequency would show phase-dependent behavior solely on the phonemic frequency (and vice versa for lexical frequency). To test this hypothesis, we used MEG in which phase-dependent categorization effects at phonemic and lexical levels of analysis could be source localized (26, 27). Earlier auditory regions such as STG are more sensitive to phoneme content, such as vowel and consonant traits, while regions higher in the auditory hierarchy, such as MTG and inferior frontal gyrus (IFG), are sensitive to lexical representation and temporal integration, respectively (26, 28–31). If phoneme or word frequency relates to phase-dependent categorization, the phase of ongoing oscillations in distinct cortical regions should bias categorization based on the level of analysis of that region. In the MEG experiment, we presented the ambiguous morphs of the consonant experiment to Dutch participants while recording their neural activity with MEG and source localizing the response to the STG, MTG, and IFG. Stimuli were not preceded by an entrainment train but were presented at random SOAs as we could extract phase from the MEG directly. Participants performed a screening for their eligibility in the MEG and MRI and gave written informed consent. The study was approved by the Ethical Commission for human research Arnhem/Nijmegen (project number CMO2014/288).

First, we looked at the overall power response in the prestimulus period (see *SI Appendix*, Fig. S3 for poststimulus responses). All analyses were based on using an array-gain beamformer which corrects for center-of-head biases without the need of a baseline (32, 33). To limit computational resources, we focused on the first component of the principle component analysis (PCA) computed over all MEG sensors targeting the bilateral region of interest (ROI) that explained the most variance in the ongoing data. In all ROIs, we found a peak in the power spectrum (peak values: 8.4 Hz, 8.6 Hz, and 8.2 Hz for STS, MTG, and IFG, respectively) across the whole prestimulus window, but this peak was weaker for the IFG (*SI Appendix*, Fig. S4). We compared prestimulus power values dependent on the response of the participant for the ambiguous stimuli but found no differences (all *P* > 0.573).

**Fig. 3.**
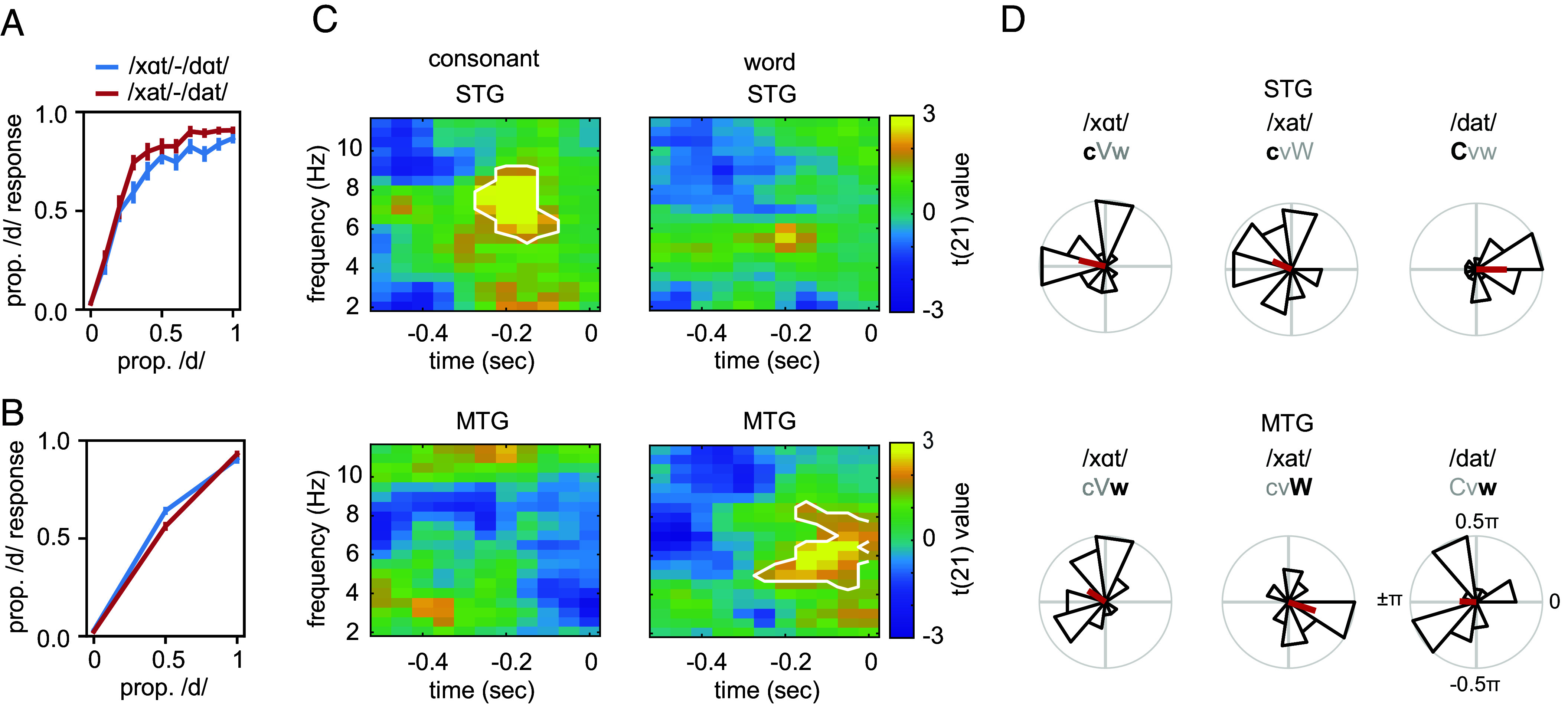
MEG results. (*A*) Behavioral responses in the MEG experiment for part one of the experiment. (*B*) Behavioral response for the main experiment. Error bars reflect the SEM. (*C*) Results of the logistic regression show show that in STG, phase determines the percept of consonant frequency (*Top*), while in MTG, phase determines the percept of word frequency (*Bottom*). White outline indicates significance (*P* < 0.05) according to the cluster-based statistical test. (*D*) Phase difference with CVW /dαt/ at the individual peak time–frequency point in *C*. In STG, words with high-frequency consonants (Cxx words) had a phase difference of 0, while words with low-frequency consonants (cxx words) had a phase difference of around π (*Top*). In MTG, high-frequency words (xxW words) had a phase difference of 0, while low-frequency words (xxw words) had a phase difference of around π (*Bottom*).

For all participants, we could individually determine the most ambiguous morph and all, but one could maintain an ambiguous percept throughout the experiment (Fig. 3 *A* and *B*; number of participants included in the analysis is 22). Data corresponding to the ambiguous sounds were then split according to the response of the participants. In the consonant contrast, we contrasted responses where the ambiguous word was interpreted as a word with a low-frequency consonant (/xαt/ [cVw] and /xat/ [cvW]) vs. a word with a high-frequency consonant (/dαt/ [CVW] and /dat/ Cvw]; averaged amount of trials included: low frequency 126.9, SD = 30.0. high frequency 189.7, SD = 27.3). In the word contrast, we contrasted responses where the ambiguous word was interpreted as a low-frequency word (/dat/ [Cvw] and /xαt/ [cVw]) vs. a high-frequency word (/dαt/ [CVW] and /xat/ [cvW]; averaged amount of trials included: low frequency 145.0, SD = 16.6. high frequency 171.6, SD = 25.9). This was done for each ROI separately. At each prestimulus time–frequency point, we performed a logistic regression with the sine and cosine of the prestimulus phase and response type (low or high trait frequency) as dependent variable per participant. Under the null hypothesis, there is no effect of phase on the response of the participant. To correct for multiple comparisons, we ran cluster-based statistics (34). For the consonant contrast, we found a significant effect of consonant frequency in the STG (Fig. 3*C*; cluster statistic: 60.1074; *P*-value: 0.028; frequency range: 5.5 to 9.0 Hz; time range: −0.25 to −0.10 s; peak t (21)-value: 4.099 at 7.617 Hz, −0.20 s), but not in the MTG or IFG (*P* > 0.05). For the word contrast, we found a significant effect of word frequency in the MTG (Fig. 3*C*; cluster statistic: 60.208, *P*-value: 0.030; frequency range: 4.4 to 8.5 Hz; time range: −0.25 to 0 s; peak t (21)-value: 3.550 at 6.445 Hz, −0.10 s), but not in the STG or IFG (*P* > 0.05). In sum, we found a double dissociation between ROI and trait type. Additionally, when directly comparing the significant consonant and word cluster within each ROI the effect was significantly larger in the STG (t (21) = 2.727, *P* = 0.018), whereas the effect of word was significantly larger in the MTG (t (21) = −3.074, *P* = 0.006). No effects were found in IFG, even after splitting up the IFG into pars triangularis, pars opercularis, and pars orbitalis.

To further evaluate the exact phase differences for each ambiguous sound, we computed the average phase at which participants heard either of the two words. Phases were extracted for each individual’s peak time–frequency point within the significant cluster (for both morphs separately). The exact MEG phase was not expected to be identical across participants as it is difficult to determine excitability levels of an oscillation from the MEG phase and individual stimulus processing times might differ. Rather, high event probabilities should be represented at the same phase, while low event probabilities are represented at the opposite phase within each participant. We took the word /dαt/, which had high frequencies on all traits (CVW word), as a reference word and took the phase difference between the average phase at which participants heard /dαt/ and one of the three words (ambiguous words perceived as /xαt/, /xat/, or /dat/). We expected that for high-frequency traits, the phase difference would be zero, while for low-frequency traits, the phase difference would be around π relative to the reference word. To test this, we performed a v-test that tests for nonuniformity with a specific direction for each of the three words per ROI. To generate a p-value that combines the three values (as we expected all three contrasts to be significant), we multiplied the three probabilities yielding the probability of all three events happening at the same time (assuming independent tests).

In STG, we found that the average phase difference for the three words /xαt/, /xat/, or /dat/ was 0.93 π, 0.87 π, and −0.002 π respectively. The three phase differences were close to the expected phase differences: the low-frequency consonants having a phase difference of π, while the high-frequency consonant having a phase difference of zero (Fig. 3*D*). The individual tests showed significance (/xαt/: vstat = 8.79, pval = 0.004; /xat/: vstat = 6.88, pval = 0.019; /dat/: vstat = 10.13, pval = 0.001, respectively) as well as the combined probability (*P* < 0.001). In MTG, the average phase difference for the three words /xαt/, /xat/, or /dat/ was 0.82 π, −0.10 π, and −0.99 π respectively. The phase differences were close to the expected phase differences: the low-frequency words having a phase difference of π and the high-frequency word having a phase difference of zero. The individual tests showed significance (/xαt/: vstat = 5.78, pval = 0.040; /xat/: vstat = 8.98, pval = 0.003; /dat/: vstat = 5.54, pval = 0.047, respectively) as well as the combined probability (*P* < 0.001). Thus, also for the individual words, the phase differences matched the neural sensitivity of the underlying region.

It is possible that due to our closed set of stimuli there is decreasing influence of lexical frequency as the stimuli are repeated many times. To test for this possibility, we compared for both the psychophysics and MEG the first and second half of the experiment with each other for the significantly found effect. For the /dat/ vs. /dɑt/ contrast, we found that there was a stronger fit for the first half compared to for the second half (*P* = 0.03; *SI Appendix*, Fig. S5). Note that while for both the first and second half there was no significant fit, the direction and pattern of the fit was the same. For all the other contrasts, no significant difference was found.

### Computational Model.

The psychophysics and MEG experiment indicate phase-dependent behavior which depend on the overall lexical and phonemic frequency of the stimulus. We hypothesized that this is a consequence of ongoing oscillatory changes in overall neural excitability together with changes in sensitivity of individual neural populations due to stronger expectations of stimulus occurrence. To test this, we created a computational model based on the Speech Tracking in a Model Constrained Oscillatory Network (STiMCON) model introduced in ref. 21 aimed to reproduce the psychophysics and MEG results. This model integrates temporal tracking together with the tracking of the content of speech. In the model, the activation of individual content representations is modulated by an oscillation set at 6.25 Hz based on ref. 21, and by the connectivity to the input structure. The sensitivity of the model is adapted by changing the threshold at which individual representations are activated. In this implementation, the activation is a function of the sensitivity, the phase of the ongoing oscillator, and the strength of the input.

In the current implementation, we introduced two different levels of analysis: a phoneme and a word level. Both levels receive input from the input level but have their own connectivity with the input and their own node sensitivity. The input is modeled as the individual words: /xat/, /xαt/, /dat/, /dαt/, and an empty word node is used for the entrainment train. For the phoneme level, we represented the phonemes /x/, /d/, /a/, /α/, and an empty phoneme node. Connectivity for the input-to-phoneme level was set to one when the phoneme was part of the input word (the entrainment stimulus was connected with a one to the empty phoneme node). The input-to-word level connectivity consists of an identity matrix (each word loads with one on the word level). Sensitivity of individual nodes to input was varied by reducing the activation threshold for the more frequent phonemes (/d/ and /α/) and words (/dαt/ and /gat/) in their respective analysis level (the base activation threshold of 1 was parametrically reduced between 0.0 and 0.5). In all simulations, we extracted the categorization response of the model by determining the deciding node that was active first after stimulus presentation (if both were active at the same time, the node with the strongest activation was chosen). See *SI Appendix*, Fig. S6 for a model diagram and *SI Appendix*, Fig. S7 for an example time course of the outcome of the model. For the two categorization options, we coded one node as a 0 and the other node as a 1. For the psychophysics experiment the outcome was deterministic (every run would give the same outcome). Therefore, if both nodes were active simultaneously at the same strength or no node was activated at all we assumed that the model would guess and set the value to 0.5. In contrast, the MEG experiment simulation was not deterministic as we simulated random phases at an individual trial level (n = 1,000). Since we estimate at a single trial level, a guess would entail a random choice of either 0 or 1 (which would be averaged across multiple trials later). For the psychophysics experiment, we assumed that the output of the model reflects the average outcome of the phoneme and word level.

**Fig. 4.**
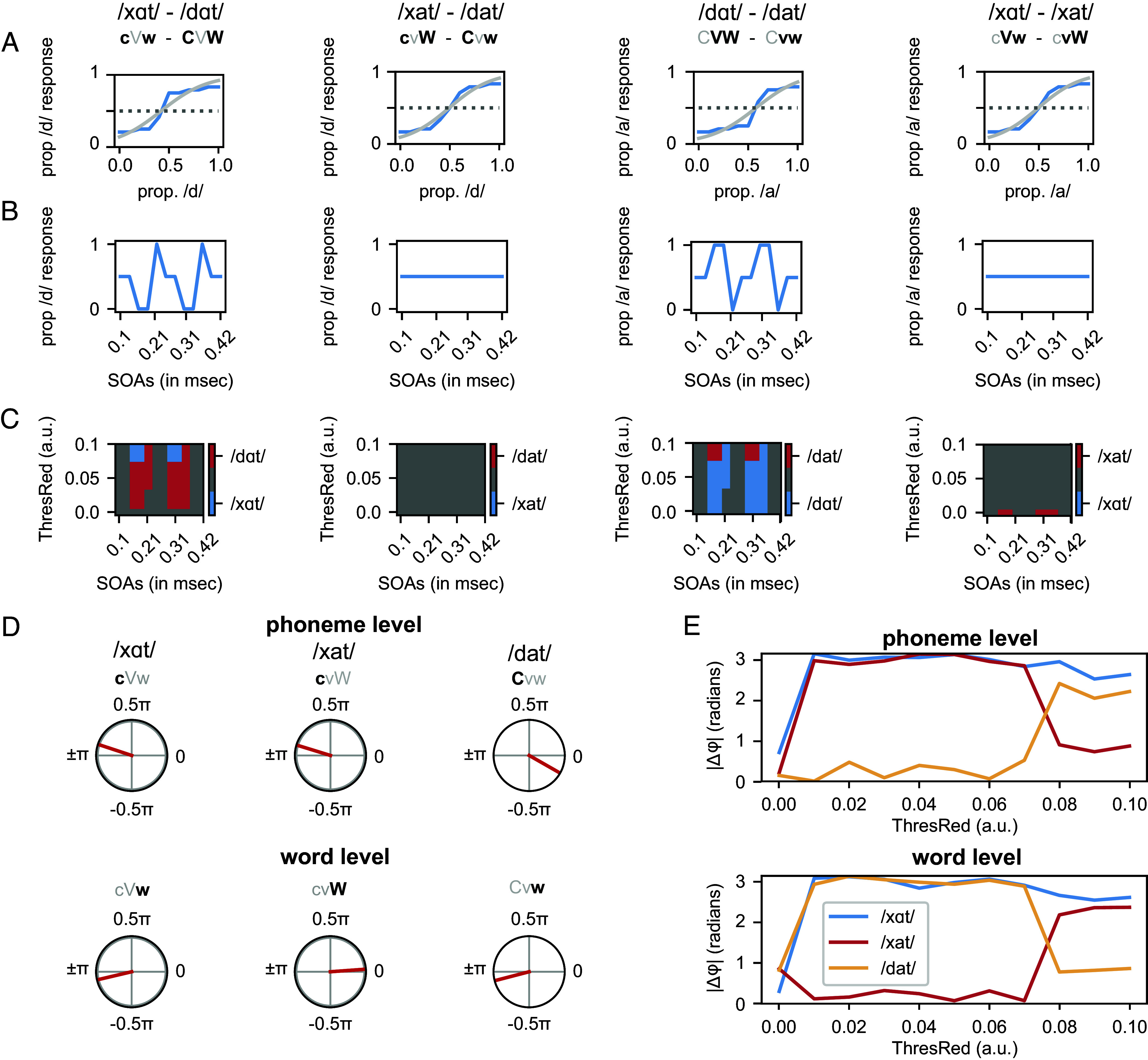
Outcome of the computational model. (*A*) Psychometric functions for the four different morph dimensions (threshold reduction = 0.3). Blue lines represent the model output; gray lines show the psychometric fit. (*B*) Response of the model to the most ambiguous morph of *A* presented at different stimulus onset asynchronies. (*C*) Response choice of the model during entrainment (as in *B*) for different threshold reduction levels. (*D*) Phase difference between the average phase of the three different response choices and /dαt/ (threshold reduction = 0.45). (*E*) Phase differences (as in *D*) for different threshold reduction levels.

We let the model run through the psychophysics and MEG experiments. In both experiments, all morphs are initially presented at random moments to generate a psychometric curve and to determine the most ambiguous stimulus that will be used for the main experiment (see *SI Appendix, SI Methods* for more details). To imitate this procedure, input was presented at different amplitude proportions of two words (e.g., for /xat/-/dat/ morph with 90% of /dat/, /xat/ was presented with an amplitude of 0.1 and /dat/ at 0.9) evenly distributed across all phase values. For each morph, we averaged the node responses of the model across the repetitions of the same morph (and across the two levels). For all word morph spectra, we could reliably fit a psychometric function and extract the most ambiguous morph (Fig. 4*A*). For the second part of the psychophysics experiment, the model was presented with an entrainment train of empty words after which we presented the most ambiguous morph stimulus at different SOAs. Results show that only for morph spectra in which the two morphed traits had opposing event probabilities or frequencies (i.e. /dɑt/ and /xɑt/ [CVW vs cVw] and /dɑt/ and /dat/ [CVW vs Cvw]), a phase-dependent categorization performance developed (Fig. 4 *B* and *C*). This phase-dependent categorization performance seemed to last across almost all sensitivities tested.

Note that the exact phase of the sine fit does not align with the computational model (compare Fig. 4 *C* and *E* with Fig. 2). However, the goal of the modeling exercise here is not to provide a quantitative estimate of the exact phase; rather to demonstrate whether the presence or absence of a phase-dependent response provides a better fit to the behavioral and neural data. In fact, estimating an exact phase would be somewhat arbitrary as its particular value depends on neural delays which are difficult to estimate properly in the current noninvasive setting. Moreover, the exact time to disambiguate /xat/ from /dat/ is also likely to be different from /dαt/ and /dat/ because the relevant feature (vowel vs. consonant) is simply at a different moment in time. Therefore, it is likely that the timing of the interpretation for the psychophysics is different for words that are ambiguous in consonants or vowels, while in the abstraction of the computational model, these factors are not included. We decided to not implement the neural delays in the model as this would complicate the model beyond what is possible to validate with noninvasive human data, and as such, we would not be able to include precise quantifications of the exact neural delays. Instead, we focus on the presence or absence of a phase-dependent response in the psychophysics and MEG data.

For the MEG experiment, phase does not have to be inferred from an entrainment train, but rather can be estimated from the recorded regions directly. To simulate this experiment, ambiguous morphs were presented to the model at random phases (repeated for 1,000 repetitions). Only the consonant morphs along the /dαt/-/xαt/ and /dat/-/xat/ spectra were used in the MEG experiment. For each of the two ambiguous morphs, the phase was extracted together with the categorization response based on the node activation of the phoneme or word level. For the main MEG experiment, we are not hypothesizing about the absolute phase, as we have no hypothesis about the exact phase (see refs. 6 and 14), but rather in relative phase difference between a more likely vs. less likely event. Therefore, we took the phase difference between the word /dαt/ which has a high frequency on all trait dimensions (CVW) and the phase of the other response options. For the phoneme level, the model showed high phase differences of around π when the ambiguous morph was interpreted as either /xat/ or /xαt/, but low phase differences of around 0 when the model interpreted the morph as /dat/ (Fig. 4*D*). In contrast, for the word level, we found π phase differences for the categorization choices /xαt/ and /dat/, and 0 phase difference for the choice /xat/. Thus, phase differences were low when both words had high-frequency traits within the level of analysis (high-frequency phonemes in the phoneme level [/dαt/, CVW vs. /dat/, Cvw] and high-frequency words in the word level [/dαt/, CVW vs. /xat/, cvW]). Phase differences were high when the words had different frequency traits (high- vs. low-frequency phonemes in the phoneme level [/dαt/, CVW vs. /xαt/ [cVw] and /xat/, [cvW] and high- vs. low-frequency words in the word level [/dαt/, CVW vs. /xαt/ [cVW] and /dat/, [Cvw]). In MEG, this pattern of results could correspond to phase differences in different neural sources that analyze phoneme- and word-level representations, respectively. These phase differences were more pronounced when the sensitivity level changes were relatively low (Fig. 4*E*).

To further investigate the outcomes of our model, we show that this model can also produce phase-dependent behavior at lower oscillatory power when representation does not reach activation thresholds at rest. In this scenario, input was modeled as linearly increasing rather than stable (*SI Appendix*, Figs. S8 and S9). Additionally, similar phase-dependent behavior is found when the phonemic level is directly connected to the word level (*SI Appendix*, Fig. S10), but it does not occur when the difference in sensitivity is removed (*SI Appendix*, Figs. S11 and S12). Finally, the model’s phase-dependent behavior works for a 5 Hz, but not 1 Hz oscillator (*SI Appendix*, Figs. S13 and S14). In sum, the current computational model makes qualitative predictions regarding the absence or presence of phase-dependent effects which are dependent on the level of analysis as well as differences in event probabilities.

## Discussion

In the current study, we used computational modeling, psychophysics, and MEG recordings to demonstrate that variations in neural sensitivity to event probability operationalized as phoneme and word frequency can result in phase-dependent perceptual categorization. We showed that ambiguous words presented at different phases, either through neural entrainment or by extracting the phase from MEG, are interpreted as one or another word depending on the time or phase of presentation. Moreover, in the MEG data, we could dissociate these effects to separate cortical regions: Phase-dependent categorization in STG depended on phoneme frequency, while word frequency modulated phase-dependent responses in MTG. The behavioral findings and the double dissociation between STG and MTG responses matched the results from a computational model that uses oscillations, together with varying neural sensitivity, to capture categorization responses. These results demonstrate that the neural phase code relies on ordering based on neural sensitivity, directly linking phase-coding to behavioral outcomes in a categorization task.

Most studies investigating the direct link between ongoing oscillatory phase and behavior have focused on assessing the role of oscillatory phase in modulating overall performance measures, such as accuracy (8, 35), detection (4, 5, 36), or reaction times (37). These studies are all based on the assumption that oscillations modulate overall firing rates and subsequent neural processing should be optimized at phases where neural excitability is high (3). We here argue that this view might be too simplistic and does not provide the full picture of the role of oscillations for neural computation. Instead of merely providing windows of processing opportunity (3, 16), previous research has proposed that oscillations provide a means to organize the complex neural dynamics by activating and thereby synchronizing neural populations at different neural phases (17, 18, 38). This organizational principle of phase coding has extensively been shown with invasive recordings in animals (39, 40). Moreover, prior computational modeling has shown the computational benefit of this organizational principle, as it effectively increases the representational space in the brain (38) and changes the formal expressive power of a system (41–43). Our study shows that this encoding process which separated neural representations by phase steers the decoding process to bias categorization toward one or another item.

It has been an open debate what are the organizational principles of phase coding, that is, what determines on which phase a neuron is activated (40). In working memory paradigms, sequence order has often been implied to be the main organizational principle of phase coding (44, 45). This is based on studies primarily in rats that show phase precession in which the order (and phase) at which neural representations are active is linked to the order of upcoming locations in an explorative maze task (39). In our study, no sequence order can be imposed. Nonetheless, we find that phase influences the behavioral decision based on the frequency of phonemes and words in the Dutch language by using psychophysics, MEG, and computational modeling. This finding suggests that sequence order is not the only principle by which neuronal representations activate at different phases of ongoing oscillations. Instead, overall event probability of words within a language modulates the neural sensitivity. Together with excitability modulations of oscillations, neural sensitivity determines on which phase a population is active (21). While the present study is focused on event probability based on the overall frequency of information in a language, we hypothesize that this finding can be extended to event probability that also depends on contextual knowledge. Evidently, contextual event probability and sequential order are related: Events that are going to happen earlier in the future are more probable in the short term. Moreover, events occurring in the near future have a higher behavioral relevance. Both probability and behavioral relevance could have a consequence for how excitable individual neural populations are. Therefore, excitability shifts, rather than solely order or event probability, could be the core principle that organizes the phase code.

The brain’s sensitivity to varying levels of event likelihood based on the statistics in the world has been shown in a plethora of studies which demonstrate that the brain is more sensitive to stimuli that are more likely (20). However, the consequence of a probability manipulation in combination with oscillatory dynamics has rarely been studied. We here provide behavioral evidence showing that event probability and oscillations together provide a phase code which activates event representations on different phases based on their likelihood. It is unknown how strongly event statistics modulate neural sensitivity. This could potentially be relevant for the behavior of a neural system as our computational model suggests a nonlinear change in phase-dependent perceptual outcomes for increasing neural sensitivities (Fig. 4*E*). Probability modulations as tested in the current study are rather static and depend on word probability in language, which has been learned over the course of one’s life. It is possible that in our current study, the effects are weaker compared to natural speech as the same closed stimulus set is repeated many times which could change how strongly lexical frequency influences word identification (*SI Appendix*, Fig. S5). It would be interesting to also investigate whether these effects are dynamic by varying event probabilities within the course of an experiment as has for example been shown for syntactic factors (46). If this manipulation also leads to similar phase-dependent categorization, phase codes would not only be adjusted solely based on long-term hard-coded changes in excitability but also based on dynamically changing excitability levels that rely on top–down feedback (21).

It has been proposed that the primary role of theta oscillations during speech comprehension involves parsing speech into separate temporal chunks (47–49). This segmentation is hypothetically done by aligning theta band oscillations with syllables in an ongoing speech stream through phase resetting mechanisms (48). In this way, one can parse and identify individual syllables and use them for higher-order linguistic operations (50). In our study, it is difficult to separate segmentation or “chunking” from any kind of process of interpretation. If a word is separately “segmented” or “chunked” by theta oscillations, the information about the phase would be lost in an identification operation. However, in our study, it is exactly the phase of the theta oscillation that determines how a word is interpreted. Note also that the reported effects are strikingly close to the 6.25 Hz frequency we found in our previous study (14). Therefore, our study shows that segmentation or chunking through oscillations cannot be treated as a wholly separate process from word recognition, because oscillations also provide a categorization mechanism alongside any potential segmentation or chunking operation (see also refs. 51–53). Additionally, it highlights how memory operations that also operate in theta ranges interact with bottom–up processing of speech.

The main feature of the model that causes phase-dependent activation of different items is the interaction between oscillatory activity that modulates population-level excitation and representation-specific excitation modulations (*SI Appendix*, Figs. S7 and S9). An additional feature of the model is self-inhibition which occurs after a period of suprathreshold activation (Eq. **1**). Inhibition ensures that a representation does not remain active for an extended period. In our main model (Fig. 4), it is necessary to have this inhibition to generate a phase-dependent effect as otherwise, the high-sensitive representation will always win over the low-sensitive representation. It is possible to generate phase-dependent effects without inhibition when changing the input (*SI Appendix*, Figs. S8 and S9 which have linearly increasing strength of sensory input instead of all-or-nothing); however, we have previously shown that self-inhibition ensures stronger phase-separation and fits better to behavioral data (see ref. 21). Besides self-inhibition, mutual inhibition of representations could also improve phase separation. In the current manuscript, we extracted the initially active node to decide the perceptual decision. Therefore, adding mutual inhibition would not have changed the effect. A final feature of our model is the frequency. We here show that the exact frequency can be variable around the range of theta (but ultimately in the model is also determined by the input parameters and activation function). Moreover, in the current implementation the oscillator aligns with the rhythmic stimulus input. Likely, there is a more complex interaction between the oscillator and the sensory input that is not modeled in our current implementation. Future studies should integrate models of oscillatory coupling together with models of phase coding to get a full picture on how oscillatory computations relevant for sensory coding and tracking.

The current model is tailored to explain a rather narrow set of observations, namely how phase-dependent categorical choices emerge from the interactions between oscillations and neural sensitivity changes that are due to event probabilities. At this stage, the model is kept small to more closely adhere to our experimental set-up and make explicit how merely changing the time of presentation can influence speech perception due to inherent coding properties of the brain. Scaling up the model is possible, but would need further experimental verification to make it more applicable to everyday circumstances. This model stands in contrast with other linguistic (54, 55) and neurolinguistic models (26, 56, 57) that try to explain speech perception or language. In most linguistic models, the full lexicon of a speaker is introduced and there are strong interactions between the levels, but neurophysiological constraints are not always considered. In recent years, models that include the role of oscillations for speech perception have emerged (e.g., refs. 47 and 58–60). Oscillations here are often a means to parse speech (60), or to organize and structure the incoming speech (50, 52), but oscillations as a memory phase code is rarely considered (18). Most speech perception models aim to align neural oscillations to the timing of the incoming speech through phase resetting of neural oscillations through oscillatory coupling with external stimuli (47, 61), or top–down temporal expectations of speech occurrences (58, 62, 63). Therefore, the phase of presentation typically does not determine the quality/choice of the perceptual interpretation like in our current model. Instead, phase has an influence on the strength of the representation in terms of accuracy improvements. While the current version of our model cannot implement a strong phase reset based on temporal expectations, models that do implement these phase resets so far cannot explain the phase-dependent categorical choices that we see here, as they do not take into account how the timing and the content of speech are dependent on each other (21, 64). Note that while there is evidence that neural oscillations do change their phase based on oscillatory dynamics mostly of rhythmic stimuli (65), there is not much evidence that there is a strong phase-reset based on top–down temporal expectations which are not rhythmic. We propose here that oscillatory resetting models must be integrated with models that incorporate a role for oscillations as a phase coder (18, 21) in order to fully account for the existing literature.

We have previously argued that during natural speech and language processing temporal information can be used to infer information content, in other words, time can be a cue for content (also see refs. 21 and 56). This time–content relation is governed by the observation that words that are more likely in the current context are uttered with shorter interword intervals (21, 66, 67). Combining this observation with theories of oscillatory tracking results in more likely words being naturally presented at earlier, less excitable phases, which we confirmed with our computational model (21). This type of phase code can aid speech comprehension: When information is ambiguous, the phase of an oscillation, and thereby the time of word presentation, can be used to determine the percept. This timing matches with the natural timing, so our model is automatically sensitive to natural temporal variation in speech that depends on the predictability of a word. Importantly, we do not argue that phase is the sole or even a primary determinant for speech identification; in natural speech, there are a plethora of acoustic and temporal cues that determine speech content. As all acoustic cues are controlled for in our stimulus set, the current study does highlight that phase is an additional cue for speech identification. Notably, our current study does put some limitations on the use of phase-dependent categorization. Our behavioral analysis shows that this phase-dependent categorization works mostly when trait features across the word are all either frequent or nonfrequent. Yet, it is not clear how likelihood information that varies based on the level of linguistic analysis interacts with timing in natural speech. To investigate this, one would need to show how top–down feedback modulates phase-dependent categorization in sentence context as top–down feedback strongly influences the sensitivity of individual word nodes as well as the timing of speech (21).

Our strongest phase effects had frequencies of 7.6 Hz and 6.4 Hz for the STG and MTG ROI, respectively (note that the peak frequency of the power in the ROIs was around 8 Hz). While for the MTG, this frequency matched with our previous finding (14), for the STG, this was slightly higher and close to alpha frequency ranges. Alpha phase (around 10 Hz) has been related to lexical decision-making such that alpha phase modulated whether participants were more likely to make a correct vs. an incorrect response to determine whether a word was a real or a nonword (68). The interpretation of these alpha findings was that there is stronger attentional selection at some high excitable phases compared to low excitable phases (3, 69, 70). It is difficult to conceive of how attention can explain why participants pick one response choice over another in our current study. Future studies are required to pinpoint the exact frequency at which our reported phase-dependent effect occurs, to highlight its links to proposed speech tracking (21, 47) and memory coding (18) mechanisms, or rather, to link it to theories of the functional role on alpha oscillations (69).

In our computational model, we postulated a separate phonological and lexical level of analysis. This is in line with neuroscientific results, which show that speech analysis is split up in separate analytical levels across the cortex (26, 27, 71). Specifically, it is suggested that STG is sensitive to phonological content, while MTG is sensitive to lexical access and word content. Note that while our current results show that STG is more sensitive to phonemic compared to word features and vice versa for MTG, we can never fully be sure that either region is exclusively sensitive to only one speech feature. Moreover, it is likely that there are subregions in the MTG and STG that are differentially sensitive to our reported effect which we did not explicitly split up further. Instead, we do report on the coefficients of the PCA components, which can hint at the centers of our effect. In our PCA components, we found for STG the strongest involvement of bilateral early auditory areas as well as anterior STG and for MTG the strongest involvement of right inferior temporal cortex bordering toward the medial temporal lobe (*SI Appendix*, Fig. S3). The early auditory regions are known to be involved in primary phonemic analysis (26, 72, 73). While anterior regions are normally reported to be sensitive to more abstract representation of words (74–76), they have also been implicated in primary phonetic analyses as we report here (77). MTG is known to be involved in word processing, although some studies suggest that the main center of this sensitivity is more superior than we find in our study (26, 75). Nonetheless, there are also various reports of activation in the inferior (73, 78) and medial (79–81) temporal cortex in lexical processing. It is known that anatomical and functional connectivity between MTG and STG operate via the early auditory cortex. There are direct connections between early auditory cortex and other regions in STG as well as connections to regions in MTG. Additionally, there are direct connections between STG and MTG (82–84). Our model currently relies primarily on the direct connections, but we also show the phase dependency effect using a hierarchical model where the phoneme layer connects to the word layer (*SI Appendix*, Fig. S10). An additional area strongly involved in the language network is the IFG. We could not find any phase-dependent categorization in the IFG. This null finding is not necessarily surprising as IFG has mostly been associated with higher-order language processes that involve temporal integration across words and syntactic analysis (30, 31, 85). IFG might simply be less sensitive to phonological or word-level likelihood differences. Alternatively, it is possible that we did not find phase-dependent effects simply because there is too much temporal variability in the IFG responses to speech. As we map phase of a cortical region directly to the onset of the presented sound, any variability will reduce the accuracy of the phase estimation in relation with the behavioral outcome. It can be expected that an area high up the processing hierarchy such as IFG has relatively high temporal variability of the neural response to speech. Therefore, as it stands, it could be that the absence of an effect in IFG is a consequence of increased noise and variability.

To conclude, we show that word categorization depends on the oscillatory phase of the neural populations in regions where lexical and phonological information effects are typically observed. The categorization bias is mediated by the trait or linguistic unit frequency that is putatively represented in the region, perhaps through variation in sensitivity of diverse neural populations. We find a double dissociation in which the phase in STG biases participants to the low or high frequent consonant percept, while the phase in MTG biases participants to the low or high frequent word percept. These results demonstrate that oscillations provide a temporal ordering of neural activation based on the excitability of neural populations. Moreover, our study highlights the role of low-frequency oscillations to organize neural activation patterns along a gradient of event probability and provide an outlook for further investigating the fundamental mechanisms that may be expressed via population rhythmic activity.

## Methods

### Psychophysics.

Morphs spectra between four different Dutch words were generated to select ambiguous sounds that were interpreted half the time as one extreme of the morph spectrum and the other half the time as the other extreme of the morph spectrum (with spectra /xat/-/xαt/, /dat/-/dαt/, /dat/-/xat/, and /dαt/-/xαt/). During the experiment, we presented a rhythmic sequence of broadband noise at 6.25 Hz before the final ambiguous word presented at variable SOA (between 0.1 and 0.42 in 12 equidistant steps). Participants were required to indicate which sound they heard.

### MEG.

Per participant, we presented ambiguous words (in between /dat/ and /xat/ or /dαt/ and /xαt/) in isolation. We investigated whether participants’ response choice would depend on the phase of the presentation. A time–frequency analysis for three regions of interest (STG, MTG, and IFG) was performed for frequencies from 1 to 15 Hz for the timepoints −0.5 up to 0 s extracting the phase of the complex spectra. Logistic regressions were performed using the sine and cosine of the prestimulus phase as independent variables. The dependent variable corresponded to a label of the response choice based on the low (labeled as zero) or high (labeled as one) frequency of the consonant or word.

### Computational Modeling.

We used a modified version of the STiMCON model (21). This model integrates temporal tracking together with the tracking of the content of speech. In the model activation of individual content, representations are dependent on the time at which that information is presented. Input activation levels at a given time (A*_l,T_*) are governed by the following function:

[1] $$A_{l,T}=C_{l-1\to l}*A_{l-1,T}+postThresAct\left(Ta\right)+osc(T),$$

in which *C* represents the connectivity patterns between different hierarchical levels (l), T the time in milliseconds, and *Ta* a vector representing the times of individual nodes within the postthreshold-activation function. Therefore, input activation is determined by activations from lower levels, an activation function, and an oscillation function. Individual *Ta* node values are set to zero as soon as activation of a node reaches activation threshold (default threshold = 1). Each node is governed by a nonlinear activation function:

[2] $$postThresAct\left(Ta\right)=\left\{\begin{array}{c} -3*BaseInhib,\;Ta\\ 3*BaseInhib,\;20\leq Ta\leq 100\\ BaseInhib,\;Ta\;<100 \end{array}\right.,$$

in which BaseInhib is a constant factor for the base inhibition level. Initiation of the inhibition function is governed by the activation threshold which was modified based on the sensitivity of the neural populations.

## Supplementary Material

Appendix 01 (PDF)

## Data Availability

MEG and behavioral data have been deposited in Radboud University Data Repository (10.34973/f4am-rd20) (86).
