# Supplementary material for: Phase-dependent word perception emerges from region-specific sensitivity to the statistics of language
Source: Proc Natl Acad Sci U S A. 2024 May 28;121(23):e2320489121. doi: 10.1073/pnas.2320489121 (PMC11161766; doi:10.1073/pnas.2320489121)
Supplement: Supplementary file 1 — Appendix 01 (PDF) [file pnas.2320489121.sapp.pdf]

PNAS

Supporting Information for

Phase-dependent word perception emerges from region-specific sensitivity to the statistics of language

Sanne Ten Oever, Lorenzo Titone, Noémie te Rietmolen, Andrea E. Martin

Email: sanne.tenoever@maastrichtuniversity.nl

This PDF file includes:

SI methods
Figures S1 to S14
Tables S1 to S2
SI References

SI methods

Behavioral experiment

Participants. In total 36 (28 female; age range = 18-40; mean age = 24.3) and 28 (20 female; age range = 20-59; mean age = 27.6) Dutch native speakers completed the session for the consonant and vowel experiment respectively. All participants reported normal hearing and did not have any history of language related disorders.

Materials. Google text-to-speech was used to utter the Dutch word *daad* (IPA [international phonetic alphabet]: /dat/; translation: *deed*) and *gaat* (IPA: /xat/; translation: *go*). We spliced the audio-file to only contain the /da/ and /xa/ parts. We max-normalized these spliced audio fragments. In praat[1] we equalized the pitch contours of /da/ and /xa/ to lie in the middle of the original pitch contours of the two sounds. For the consonant manipulation we morphed the two sounds together by taking a weighted average of the two audio fragments in 11 spaced steps. Note that this step is different than in our original study[2] in which we changed the formants directly, but it was necessary as we could not achieve a guttural /x/ made in Dutch by using a formant change only. While for syllable perception this procedure is not a problem (even though the sound is then closer to a /ga/ than a /xa/), for the chosen words a guttural /x/ is needed to understand the words. For the vowel manipulation, we subsequently changed the temporal modulation (for all 11 morphs) of the original /a/ sound to be 0.75 of the original duration using PSOLA[3] (which can maintain pitch while changing temporal rate). Then we changed the spectral content of the second formant in 11 steps from 1300-1700 Hz during the vowel utterance using the burgs LPC method[4]. This morph generates the phoneme /ɑ/. We again max-normalized the output of these morphs. We spliced from the original /dat/ sound fragment the /t/, shortened it to 0.9 of the original length (to improve the sound audibility) and concatenated the /t/ at the end of all created morphs. The amplitude of the last 0.2 seconds was linearly dampened. This whole procedure created a total of 11×11 morphs, morphing between the four extreme sounds /xat/, /dat/, /xɑt/, and /dɑt/. Note that from all these morphs we only used a total of 11×4–4 sounds, that is, the sounds at which either the consonant or vowel was at its most extreme value. All sounds had a duration of 420 ms.

We choose these words as they are dissociable in vowel, consonant and word frequency (see table 1). To verify that these words indeed had varying vowel, consonant, and word frequency we counted the number of the vowels, consonants, and words in the manually annotated part of the Corpus Gesproken Nederlands (CGN; [Version 2.0.3; 2014]). Frequency was determined by dividing the number of occurrences by the total amount of annotated words. As this results in very low numbers, the proportion is represented on a log scale. For the phonemes, we additionally repeated this analysis separating the phonemes per position in a word position (SI Appendix Fig. 1).

For the psychophysics experiment, we presented a rhythmic sequence of broadband noise at 6.25 Hz before the word. Broadband noise consisted of 50 ms (with 5 ms linear amplitude ramp

up and down) between 1.1 and 3.1 kHz. Sequences lasted randomly 2, 3, or 4 seconds. Stimulus onset asynchrony (SOAs) of the word relative to the final noise in the sequence was set to be between 0.1 to 0.42 in 12 equidistant steps (covering two cycles of 6.25 Hz).

Procedure. All procedures were done online using Django web development, running under Apache. Participants were instructed to use headphones for the experiment and sit in a quiet room. Of course because of the online setting, we could not verify this. In the first part of the experiment, we determined the most ambiguous stimulus as the morph for which participants heard on of the two extremes of the morph 50% of the time. In both experiments, this entails two morph spectra: for the consonant experiment the /dat/-/xat/ and /dɑt/-/xɑt/ spectra; for the vowel experiment the /dat/-/dɑt/ and /xat/-/xɑt/ spectra. To do so, we presented all 22 morphed sounds for the respective experiment and participants had to indicate what word they heard. A trial consisted of a silent period of 0.5 seconds followed by the presentation of the audio fragment. 0.25 seconds after the sound, participants viewed the response options and could indicate via a button press which sound they heard. Participants received two response options corresponding to the two extremes of the spectrum to which the sound morph belonged to. In total, each sound was presented 12 times, corresponding to 264 trials divided into two blocks. In total, the first part lasted about 8 minutes. Immediately after this part for both spectra a psychometric logit function was fitted, and the most ambiguous sound was determined.

The second, main, part of the experiment consisted of 14 blocks in which we presented the rhythmic sequences with the final words. In total we presented 648 experimental trials: nine repetitions, three sequence lengths, twelve SOAs, and two sound types. We added 5% of filler trials consisting of the extreme sound types (at random sequence length and SOAs) resulting in a total of 680 trials. These trials were added to test that participants were performing the task and not randomly pressing buttons and to make sure that in some instances there was also a clear correct answer. Again, after the button press there was an interval of 0.5 seconds. Throughout the experiment we adapted the ambiguous sound when participants heard the same sound too often in a row. Specifically, if participants categorized the ambiguous sound as the same word for 10 times in a row (for each spectra), we adjusted the ambiguous sound one morph step away from the perceived word.

Behavioral analysis. For the first part of the experiment, we fitted a psychometric curve using the curve_fit function in the scipy toolbox in python and extracted the most ambiguous stimulus for each participant. We had difficulty to ensure that participants maintained an ambiguous percept either during the psychometric determination or during the main experiment. We therefore had to exclude quite a few participants from the analysis. This is likely due to the online procedure that needed to be done during the COVID pandemic to which we had to rely on the audio of the participants at their home situation. As the experimental results hinge on having an ambiguous percept, we needed to exclude participants who could not maintain an ambiguous word perception

throughout the experiment. During the first part of the experiment, we could not fit an ambiguous sound that stood in between the 10th and 90th percentile of the morph spectrum for 7 and 4 participants for consonant and vowel experiment, respectively. Also, during the main experiment, 6 and 11 participants reported low perceived differences between the two unambiguous words in the corresponding spectrum, respectively (under 20% difference between the answers to the two unambiguous words; likely due to a failure to comply with the task or difficulties with the task itself). An additional 5 and 1 participants reached morphs outside the 10th and 90th percentile ranges for more than half the duration of the main experiment, respectively. This ended us with 18 and 12 participants for the consonant and vowel experiment, respectively. Trials in which morph estimations were outside of the 10th and 90th percentile ranges were excluded from the analysis.

For those participants who met our criteria, we created a time course across the twelve SOAs used. For each participant we subtracted the mean of the time course. Then we averaged all time-courses and fitted a 6.25 Hz sinusoid to the data (with varying amplitude and phase) and extracted the explained variance. We generated a null distribution by randomly permuting (n = 10,000) the average time course and refitting the sinusoid. One-sided p-values were extracted by comparing the proportion of the observed explained variance with the explained variance of the null distribution. As these comparisons were pre-planned, we did not further correct for multiple comparisons.

MEG experiment

Participants. 23 Dutch native speakers (13 females; age range: 18-59; mean age = 34.3; one author participated as well) participated in the study. 22 were right-handed (one reported no preference in hand). All reported normal hearing, had normal or corrected-to-normal vision, and did not have any history of dyslexia or other language-related disorders. All participants were reimbursed for their participation. One participant was excluded for not maintaining an ambiguous percept throughout the experiment.

Procedure. Just as the behavioral experiment, the MEG experiment consisted of two parts. In the first part, we repeated the presentation of the psychometric function to determine the most ambiguous sounds. After the sound was finished the response options were immediately shown. The next sound was presented at random interval between 0.5-1.5 seconds after the response. In the MEG experiment, we only used the consonant morphs. During the main part of the experiment, we presented 50% of the time ambiguous (n = 160 per spectrum) and 50% of the time non-ambiguous sounds (n = 80 for each extreme per spectrum). All trial types were presented in pseudo-random order. After sound presentation, there was a 1 second interval before the sound options were shown. The next sound was presented at a random interval between 2 and 4 seconds after the response. After 80 sounds participants had a break. The response options of participants (pressing left or right for /d/ vs /x/, respectively) were switched halfway through the experiment to

ensure that the effects were not due to motor plans. Half of the participants started with /d/ as the left option and the other half with /x/ as the left option. During the experiment, the morph was changed if the participant reported the same percept for the ambiguous sound within one spectrum for 10 consecutive answers. At the end of the experiment, we collected an auditory localizer (data not analyzed here) and a scalp digitization using the Polhemus Fastrak digitizer. All stimulus presentation was programmed in Psychtoolbox[5] and run in the linux environment.

MEG pre-processing. Surface-based source models from the MRI were made using grid points that were defined on the cortical sheet of the automatic segmentation of freesurfer6.0 [50] in combination with pre-processing tools from the HCP workbench1.3.2 [51] to down-sample the mesh to 4k vertices per hemisphere. The MRI was co-registered to the MEG by using the previously defined fiducials as well as an automatic alignment of the MRI to the Polhemus headshape using the Fieldtrip20211102 software [52]. Head models were based on the SPM segmentation incorporated in Fieldtrip. Regions of interest (ROIs) were the superior temporal gyrus (STG), the middle temporal gyrus (MTG) and the inferior frontal cortex (IFG). Parcellations were based on the Freesurfer parcellations.

Preprocessing of MEG involved epoching the data both between -2 and 0 seconds and -1 to +1 seconds relative to sound onset. This separate epoching was necessary to ensure that for the pre-stimulus analyses no data from the post-stimulus interval could leak into the pre-stimulus interval due to filtering during preprocessing. Data was then padded for 0.5 seconds at the end of the epoch with the last value of the epoch. Data was low-passed at 100 Hz and DFT notch filters were applied at 50, 100 and 150 Hz. Then, data was down-sampled to 300 Hz and the padded interval was removed again. ICA was performed to remove heartbeat-related signals and eye blinks and movements. On average 4.3 components (range: 3-6 components) were removed from the analysis. After that, trials with excessive noise were removed via visual inspection with an average of 12.7 removed trials (range: 4-22 trials). We calculated a common spatial filter using lcmv filter based on the post-stimulus data with a lambda of 5%. Many spatial filters have a center of the head bias, resulting in stronger activity in the center compared to the cortical surface[6]. This bias is often counteracted by having a clear baseline period for each trial to which the data is referenced to. However, for our analysis no clear baseline period can be defined as we were interested in the pre-stimulus period. Therefore, to counteract the center of head bias we used an array-gain beamformer which normalized the spatial filter[6]. This filter was applied to all single trial estimates of the pre-stimulus data. To extract a single time course representative of our ROIs we extracted the first PCA for each ROI.

MEG analysis. We performed a time-frequency analysis on all source trials using a wavelet approach extracting frequencies from 1 to 15 Hz in steps of 0.5 Hz at widths matching 700 milliseconds for the timepoints -0.5 up to 0 seconds in steps of 0.05 seconds extracting the phase

of the complex Fourier spectra. Data corresponding to the ambiguous sounds were then split according to the response of the participants based on two different contrast binning:

1) Consonant frequency binning: responses where the ambiguous word was interpreted as a word with a low frequency consonant (/xɑt/ and /xat/) versus a word with a high frequency consonant (/dɑt/ and /dat/).
2) Word frequency binning: responses where the ambiguous word was interpreted as a low frequency word (/dat/ and /xɑt/) versus a high frequency word (/dɑt/ and /xat/).

For these two contrasts, low frequency traits were labeled with a zero and high frequency traits with a one. A logistic regression was performed using the sine and cosine of the pre-stimulus phase as independent variables and the response of the participant as dependent variable for all time and frequency points. To compare these values across participants, we performed statistics using the inverse of the normative cumulative distribution based on the p-value of the regression of the individual participants (also see[7]). Group statistics were performed by statistically testing these z-values against zero using a one-sample t-test. To control for multiple comparisons across all time-frequency points we performed cluster-based permutation tests[8].

To further inspect the effect of the two contrasts we split the data based on all four possible response options. Then, we extracted for each participant and for each of the possible perceived words the average phase at which participants reported perceiving that specific word. We calculated the phase difference between the average phase at which participants perceived the word /dɑt/ and the other three options. The logic of this analysis was as follows: /dɑt/ has high frequency features for all investigated feature dimensions. Words that also have a high frequency content should therefore show a phase difference of zero with /dɑt/, but words that have a low frequency content should show a phase difference of π with /dɑt/. We statistically tested whether the phases were non-uniform around the expected phase using the v-test statistic[9,10]. This test will show a significant effect only when the data is both non-uniform and the phase is around the expected phase.

For the power analysis, we used the same wavelet approach as for the time-frequency plots (Figure 4C) and converted the data in z-scores across the whole time-frequency window. For the power spectra (Figure 4B), we cut the data from -1 to 0, padded the data out to 5 seconds and extracted the power using Hanning tapers.

Computational modelling

We used a modified version of the Speech Tracking in a Model Constrained Oscillatory Network (STiMCON) model[11]. In this model, a population of neural nodes is modulated by an oscillation and individual nodes are additionally modulated based on their connectivity pattern with sensory input. Input activation levels at a given time ($A_{I,T}$) are governed by the following function:

$$A_{l,T} = C_{l-1 \to l} * A_{l-1,T} + postThresAct(Ta) + osc(T) \quad (1)$$

in which C represents the connectivity patterns between different hierarchical levels (l), T the time in milliseconds, and Ta a vector representing the times of individual nodes within the post threshold-activation function (see online methods). Input activation is thus determined by activations from lower levels as well as an activation function and an oscillation function. Individual Ta node values are set to zero as soon as activation of a node reaches activation threshold (default threshold = 1). This activation function first ensures non-linear supra-threshold activation after which the node is temporally inhibited. The oscillation function in our implementation is fixed to a frequency of 6.25 Hz (based on our previous findings[2]). Each node is governed by a non-linear activation function:

$$postThresAct(Ta) = \begin{cases} -3 * BaseInhib, Ta \\ 3 * BaseInhib, 20 \leq Ta \leq 100 \\ BaseInhib, Ta \; > 100 \end{cases} \quad (2)$$

in which BaseInhib is a constant factor for the base inhibition level (set to -0.2, same as in [11]). Initiation of the inhibition function is governed by the activation threshold (by default set on 1, but varies with neural sensitivity, see main text). First, this function creates suprathreshold activation after which nodes are inhibited. The oscillatory function is as follows:

$$osc(T) = Am * \cos{(2\pi\omega T + \phi)} \quad (3)$$

in which Am is the amplitude of the oscillator (set to 1.5), ω the frequency (set to 6.25Hz in accordance with [2]), and φ the phase offset (variable). For the psychophysics experiment the phase offset is equalized with the phase of the stimulus input. In the model, sensory input is directed to two different levels of analysis, a phonetic level and a word level of analysis. Sensory input itself is modelled as a step function lasting 50 ms. The maximum strength of the sensory input depends on the morph level presented (see main text).

Figure S1 to S14

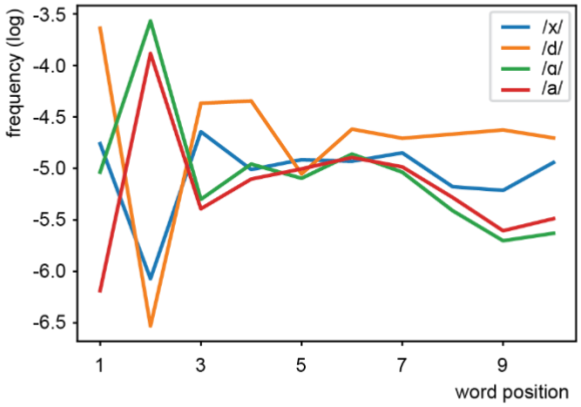

Fig. S1. Position dependent frequency of the phonemes used in this study.

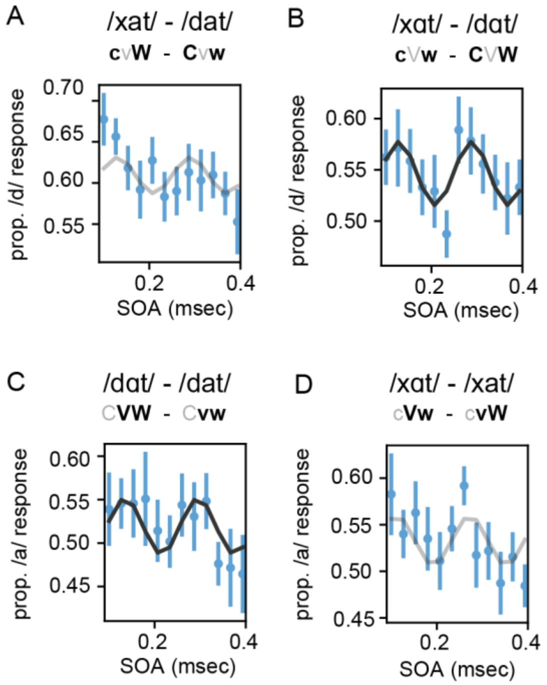

Fig S2. Behavioral results without demeaning. Conventions are the same as in Fig. 3.

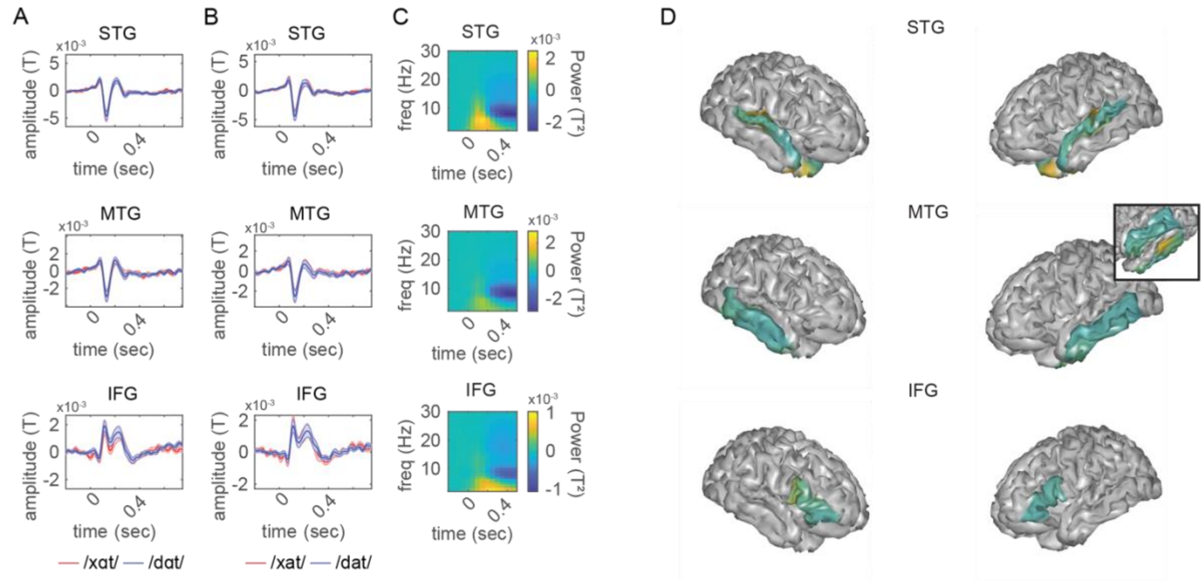

Fig. S3. Responses in regions of interest. A) Response to ambiguous stimulus /xɑt/-/dɑt/. B) Response to ambiguous stimulus /xat/-/dat/. C) Time-frequency response averaged across all stimuli. D) Average PCA coefficients across participants for the first principle component for each of the ROIs. Yellow indicates a stronger average coefficient (but exact number is arbitrary). For MTG we added an extra differently oriented image to show the strong right inferior PCA coefficient. STG = superior temporal gyrus. MTG = medial temporal gyrus. IFG = inferior frontal gyrus.

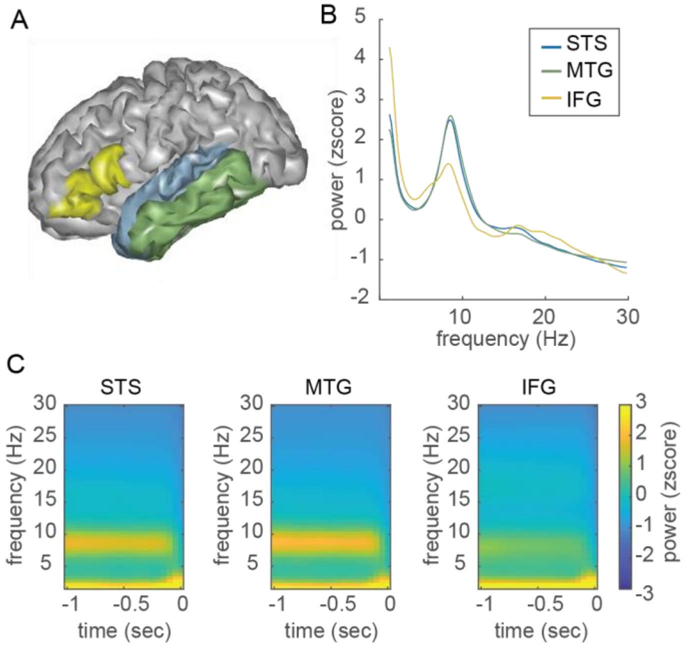

Fig. S4. Pre-stimulus power in regions of interest. A) The three regions of interest. B) Power spectra averaged for the -1-0 sec time window averaged across the two ambiguous stimuli and response choices. C) Time-frequency response averaged across the ambiguous stimuli and response options. Note that data is padded from time point 0 on (explaining the sharp power drop around zero).

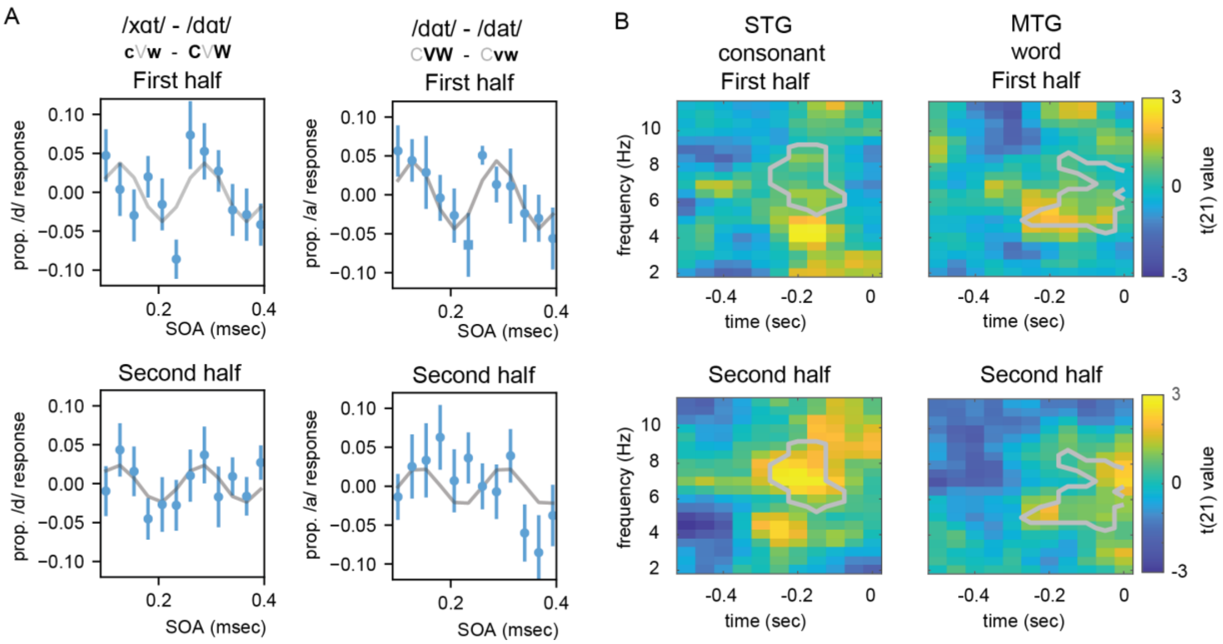

Fig. S5. Split of data in first and second half for psychophysics (A) and the MEG (B). In A) the gray line indicates the best fit. In B) the gray contour indicates the original significant cluster region.

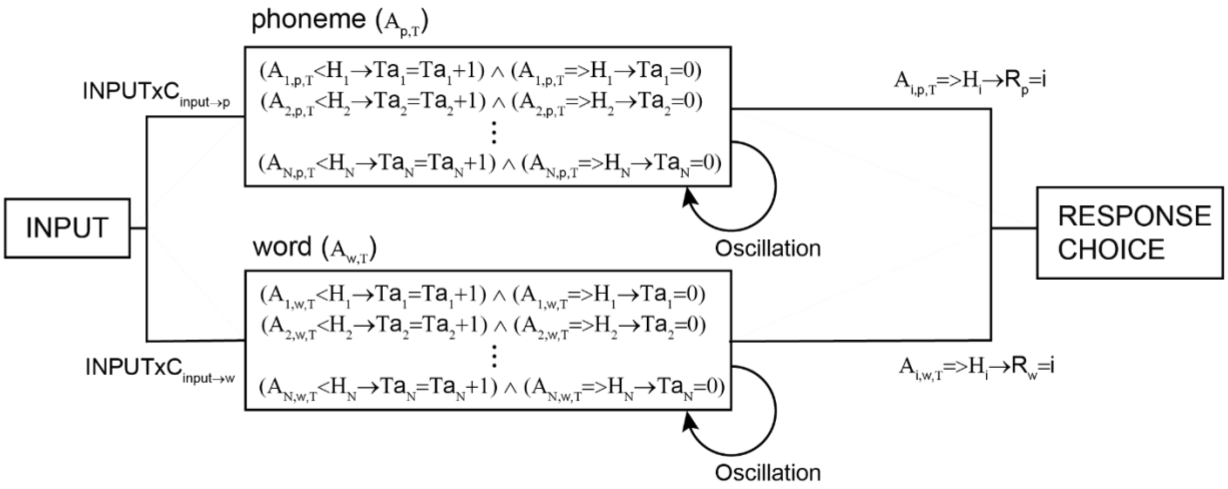

Fig. S6. Schematic diagram of the model. Input is split to either the phoneme (p) or word (w) level. Activation (A) at time point T depends on the input strength and connections (C) with the respective levels, a common oscillator, and the postThresholdActivation function (not visualized). As soon as the activation of a node in the phoneme or word level reaches it activation threshold (H) Ta is set to zero for that node and the postThresholdActivation function is reset. Response choice (R) is defined as the first node that reaches activation. For the psychometric experiment simulation, the response choice is the average of the two options. For the MEG simulation the responses choice is the output of the two respective levels.

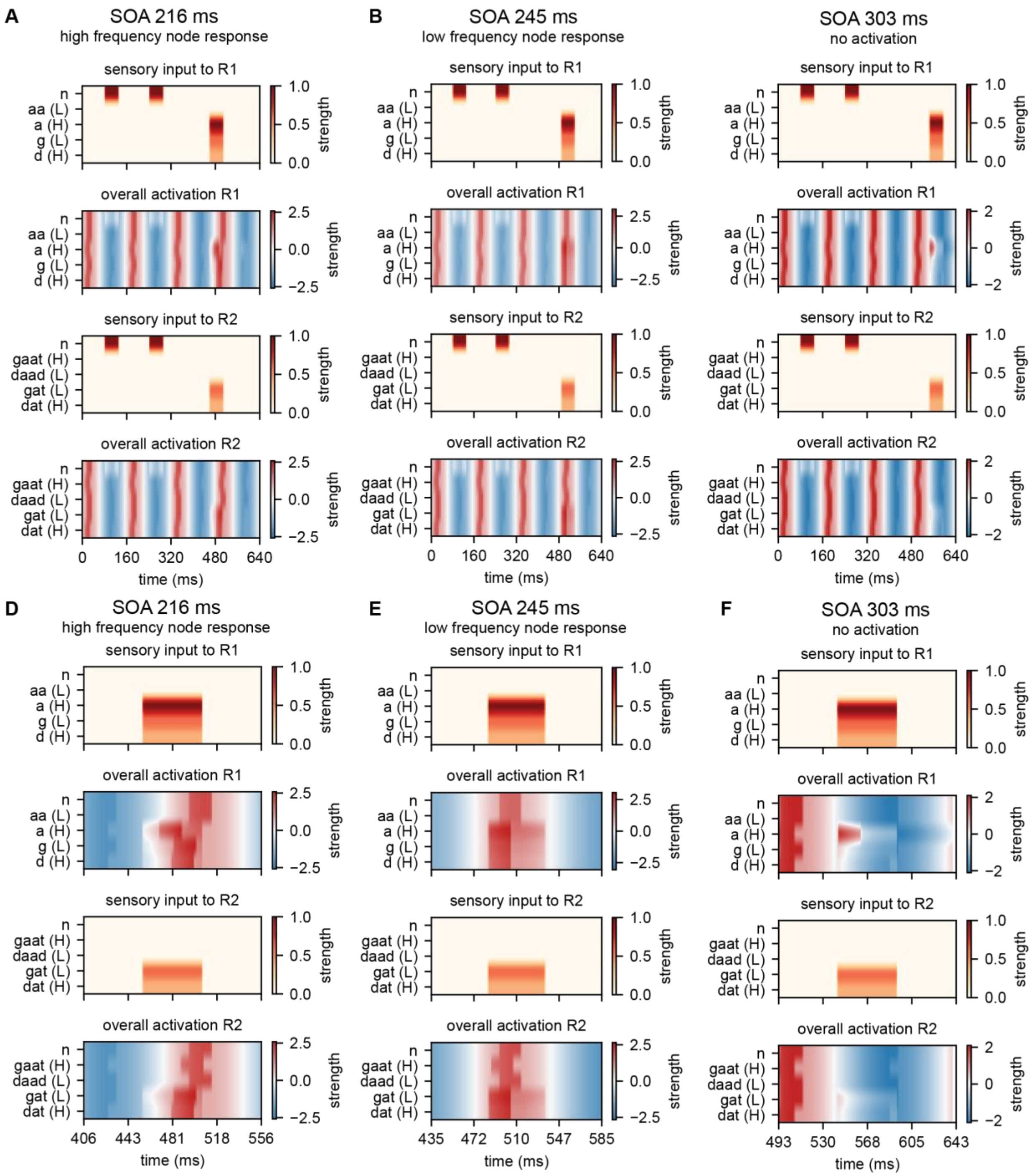

Fig. S7. Time courses of example simulation corresponding to the model parameters in Fig. 4. Simulations are run with a threshold reduction of 0.3. A-C show activity of the phonetic (R1) and word (R2) level of the model at different delays. D-F show the same time courses but zooming in on the area highlighted in A-C respectively. L and H stand for low and high probability event respectively. N is a neutral stimulus. SOA = stimulus onset asynchrony relative to the neutral entrainment input.

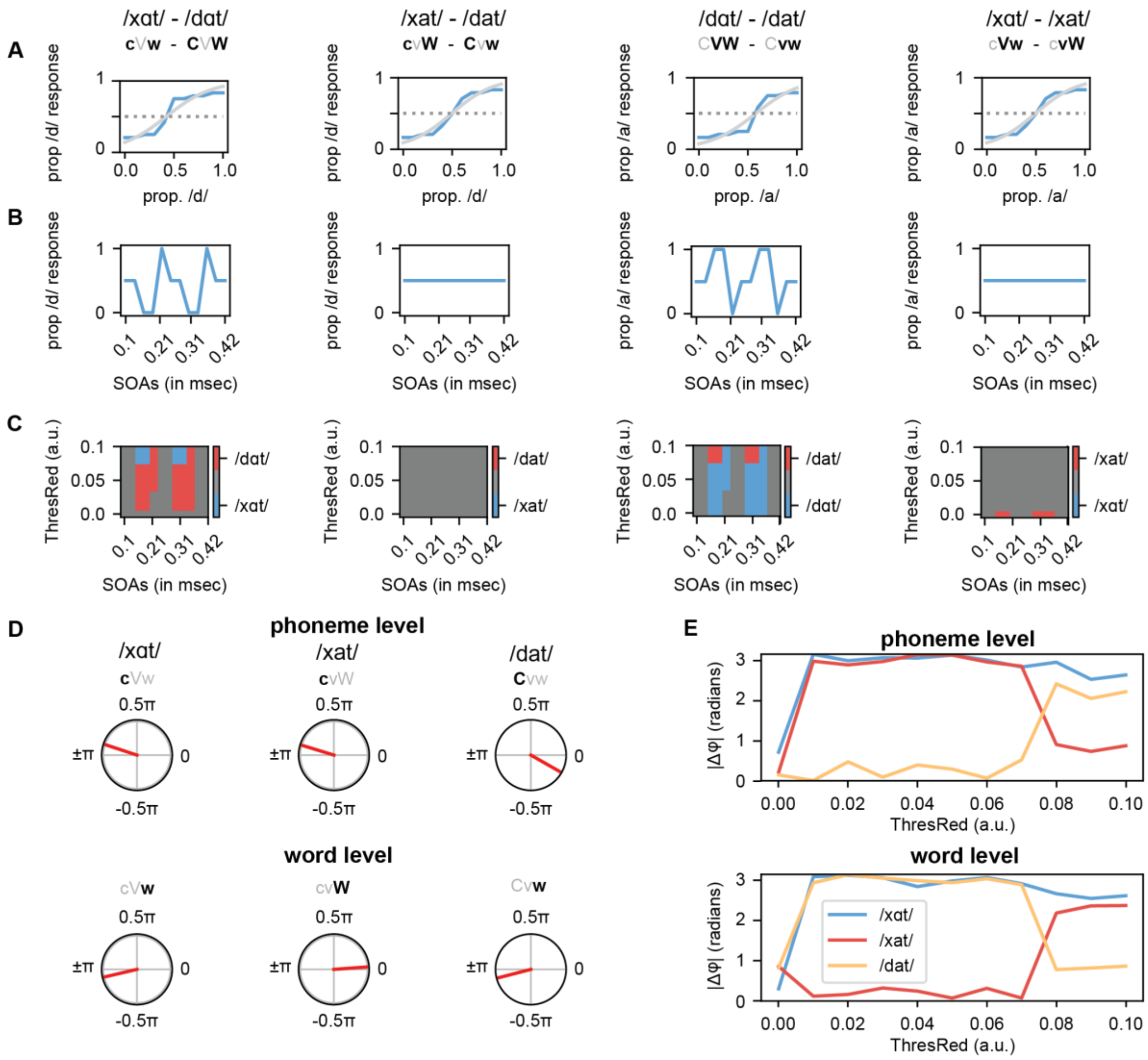

Fig. S8. Outcome of the computational model with linear increasing input and lower power. A) Psychometric functions for the four different morph dimensions (threshold reduction = 0.09). Blue lines represent the model output; grey lines show the psychometric fit. B) Response of the model to the most ambiguous morph of A presented at different stimulus onset asynchronies. C) Response choice of the model during entrainment (as in B) for different threshold reduction levels. D) Phase difference between the average phase of the three different response choices and /dɑt/ (threshold reduction = 0.07). E) Phase differences (as in D) for different threshold reduction levels. Compared to the model in Fig. 4, the oscillatory power is set to 1 (instead of 1.5) and the threshold reduction levels are weaker. Input here is linearly increasing and lasts half a cycle.

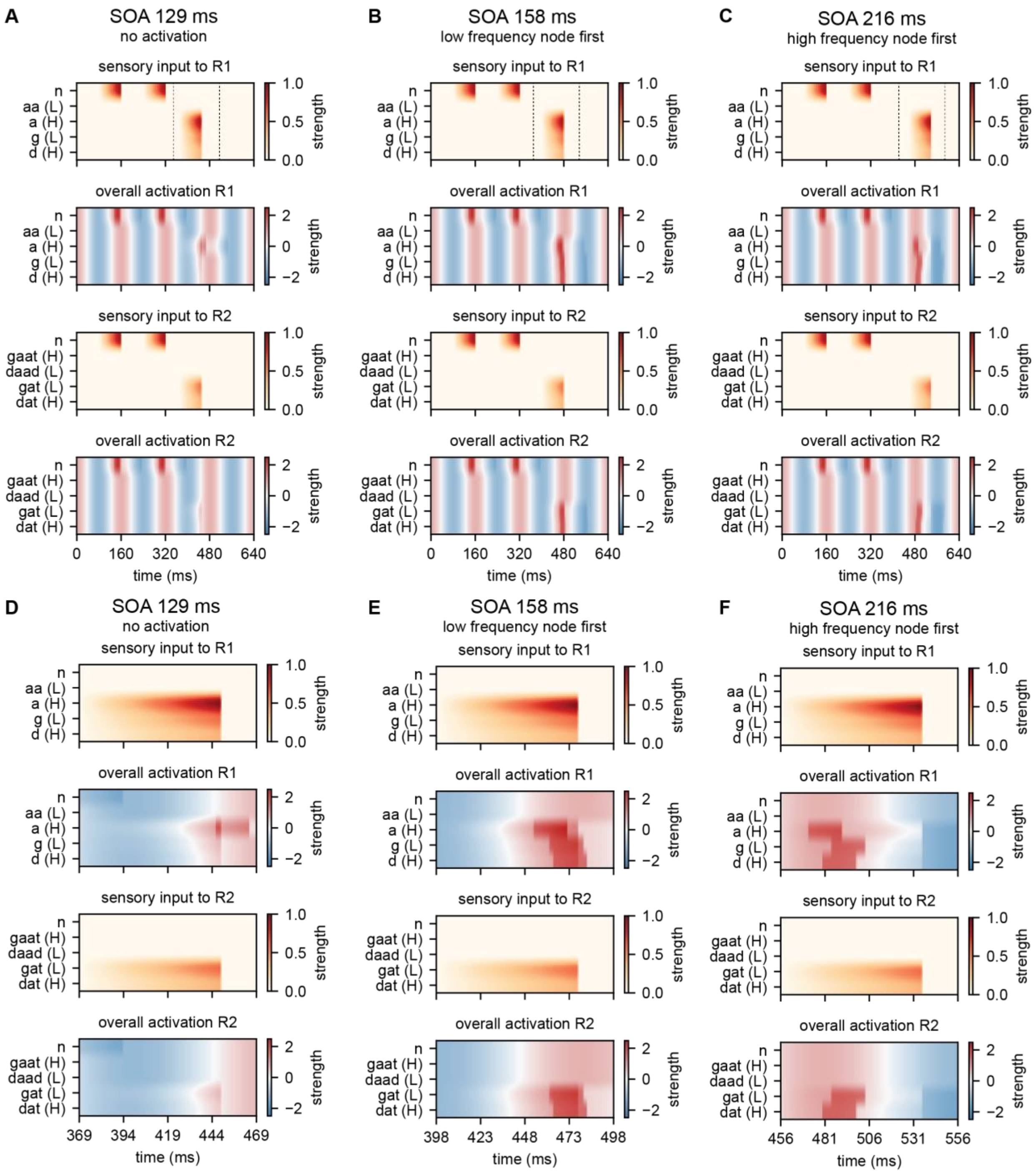

Fig. S9. Time courses of example simulation corresponding to the model parameters in SI Appendix Fig. 8. Simulations are run with a threshold reduction of 0.09. A-C show activity of the phonetic (R1) and word (R2) level of the model at different delays. D-F show the same time courses but zooming in on the area highlighted in A-C respectively. L and H stand for low and high probability event respectively. N is a neutral stimulus. SOA = stimulus onset asynchrony relative to the neutral entrainment input.

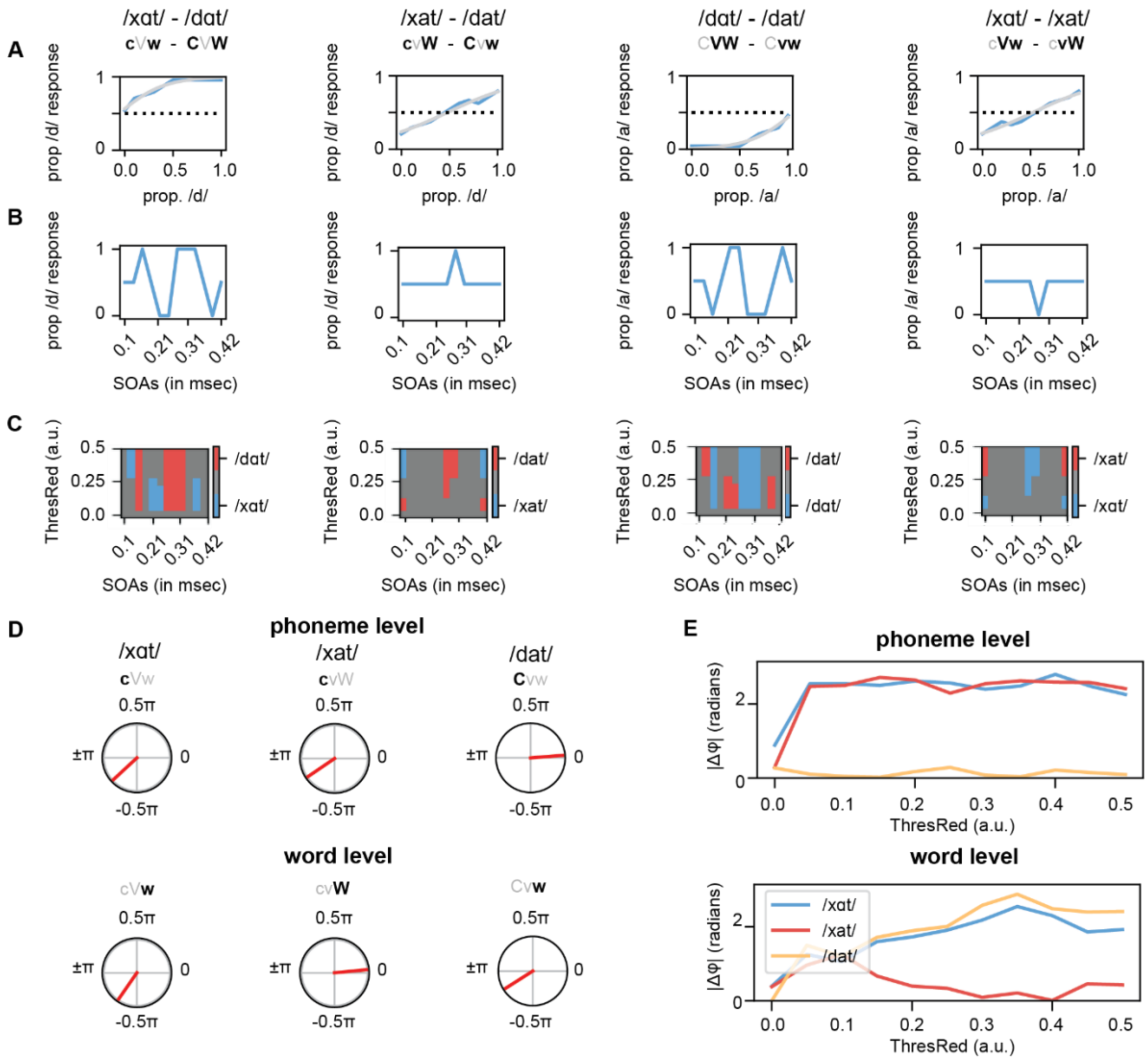

Fig. S10. Outcome of the computational model directly connecting the phoneme with the word level. In this model the phonetic level links to the word level. The input only connects to the phonetic level and this level links to the word level (in which phonemes part of the word link at a 0.5 connection strength to the words they are connected to, for the neutral phoneme the connection strength is 0.2 across all words). The outcome for the psychometric curve and the entrainment is based on the word-level activations. With this model, we can replicate the sinusoidal pattern in the behavioral responses as well as the phase opposition in the MEG, but do see some spurious response choices for /xat/-/dat/ and /xɑt/-/xat/. This is a consequence of the phonetic level response influencing the word-level response. Moreover, the psychometric functions do not fully match the found psychometric functions. Because the response choice is mostly driven by the word-level, to make a stimulus ambiguous it in this model has to be far away from the high frequency word option. So for that part the psychometric function is better fitted with a model where response options are weighted by both the phoneme and word level representation.

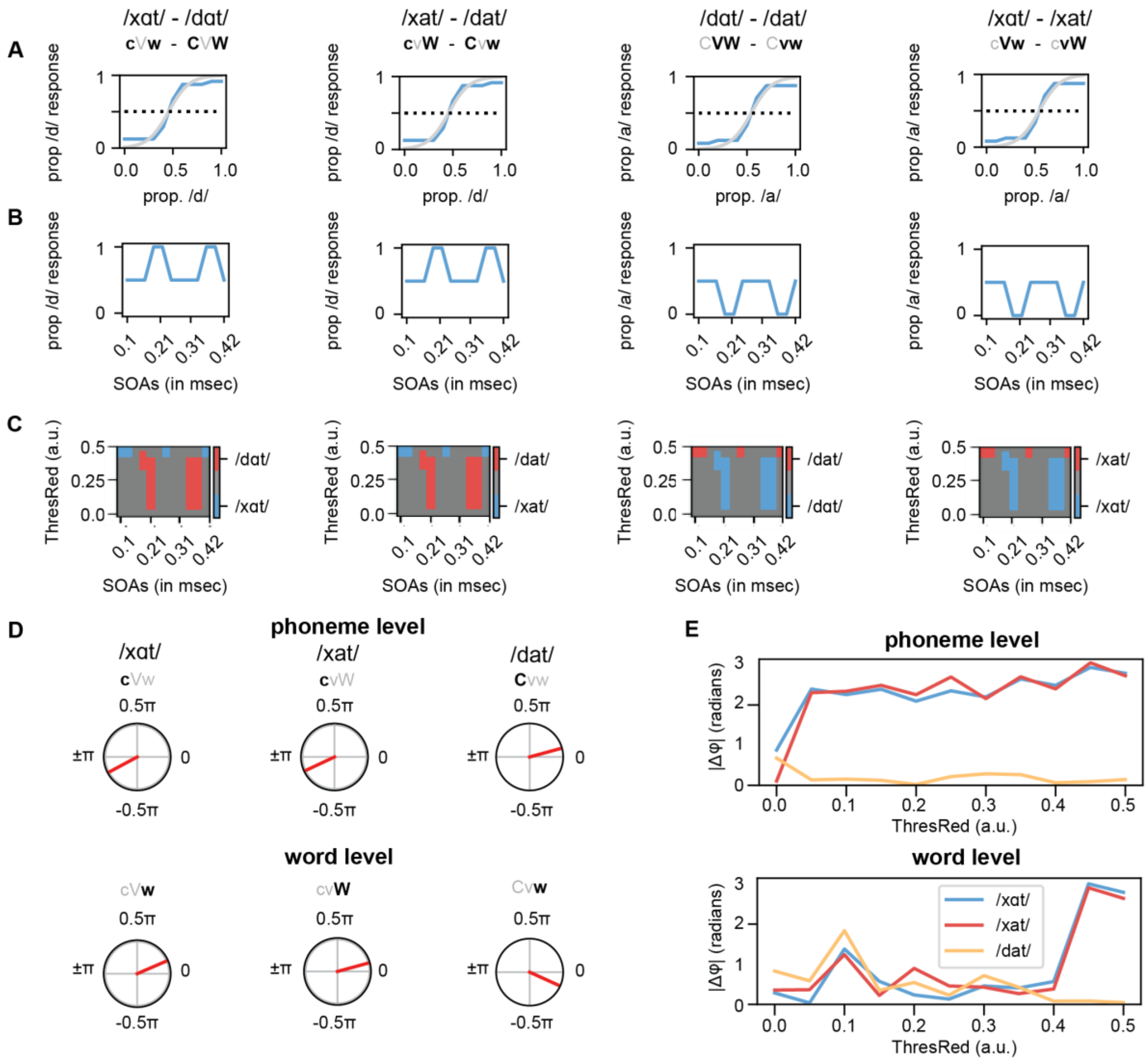

Fig. S11. Outcome of the computational model with no difference in the word level event probabilities. Conventions are the same as in Fig. 4. Note that phase-dependent effects now solely depend on the phoneme level probability differences.

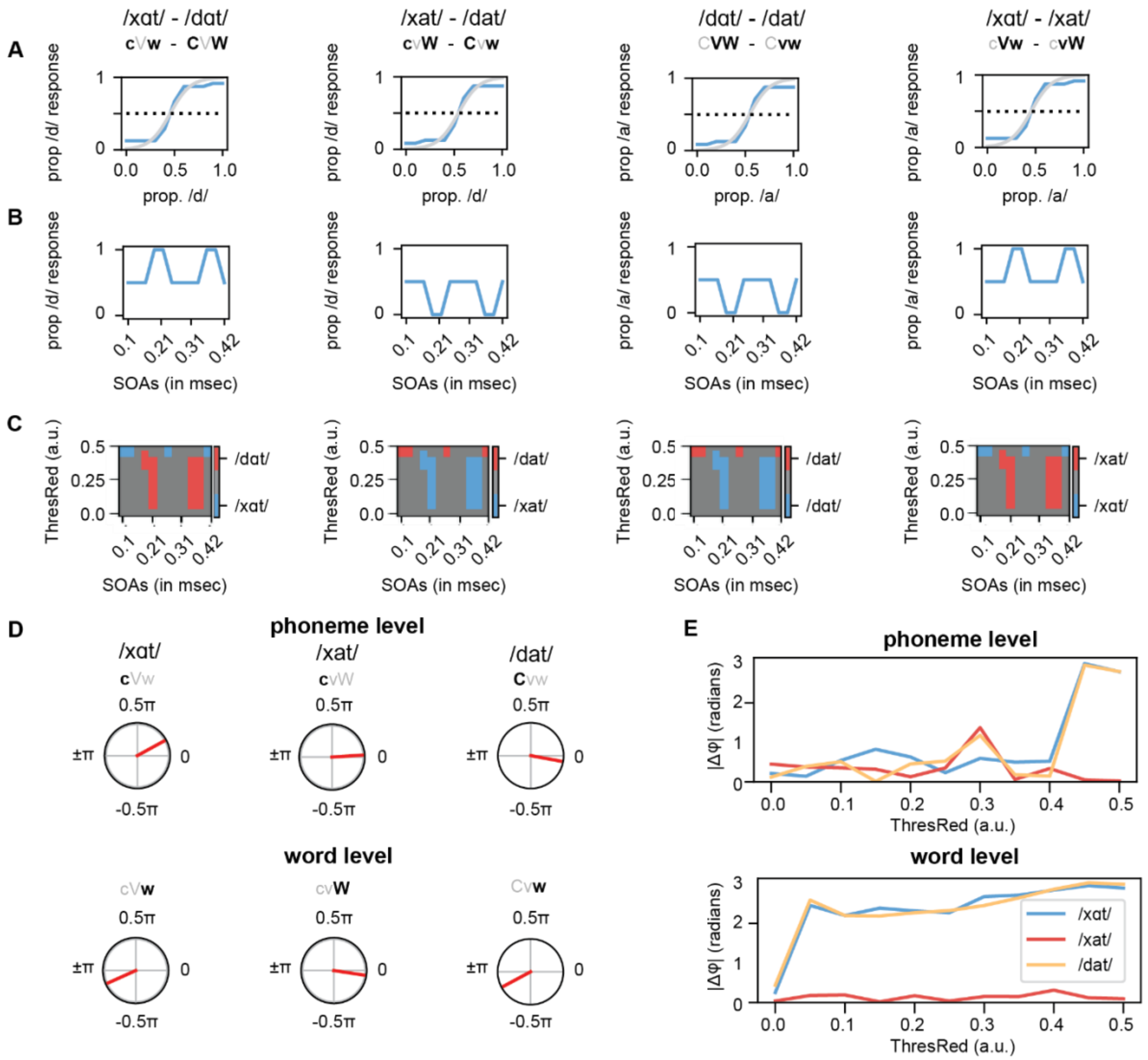

Fig. S12. Outcome of the computational model with no difference in the phase level event probabilities. Conventions are the same as in Fig. 4. Note that phase-dependent effects now solely depend on the word level probability differences.

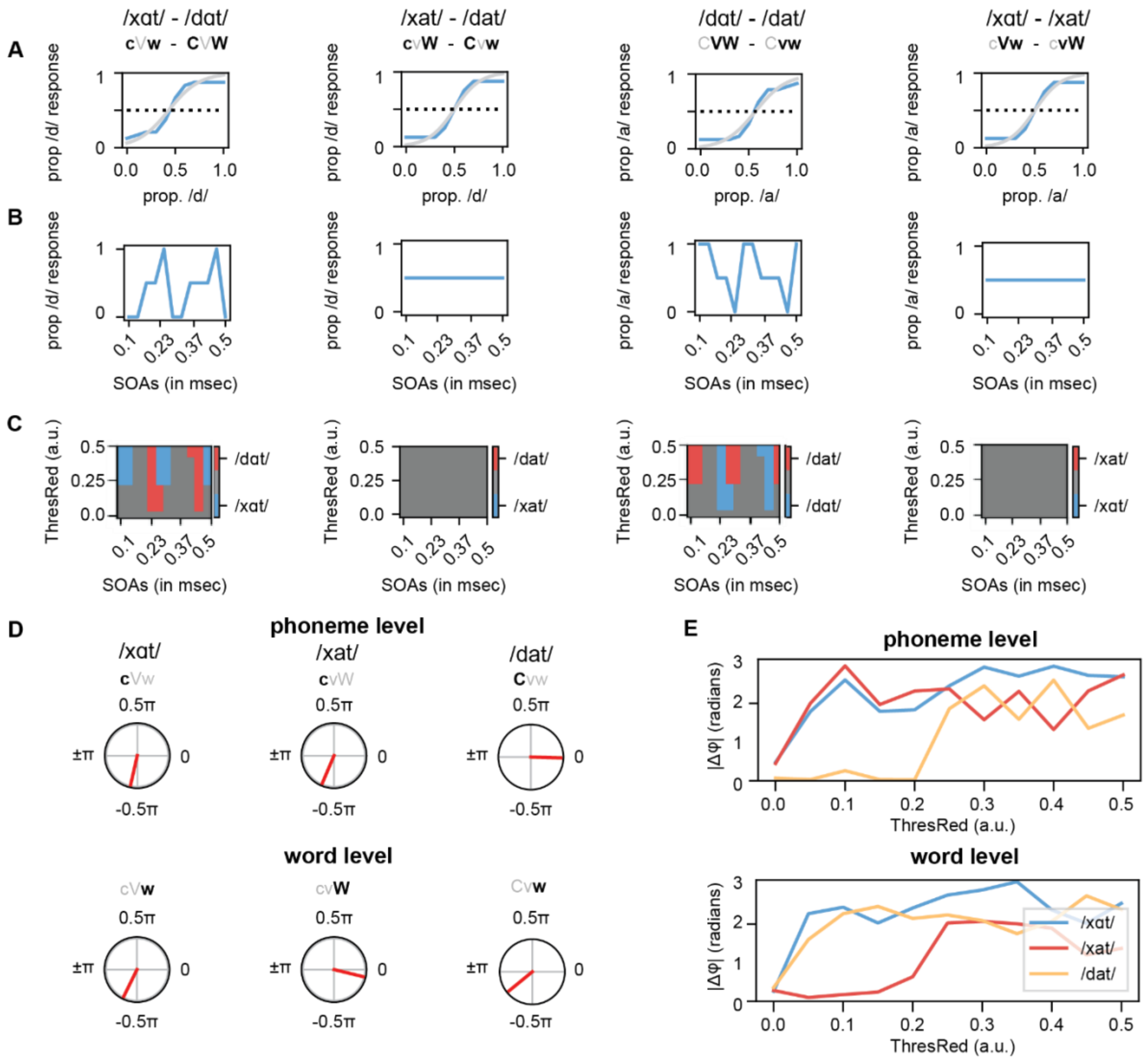

Fig. S13. Outcomes of the computational model when using a 5 Hz oscillator. The entrainment frequency is also adjusted, but the duration of the stimuli and activation function remained the same. All conventions are the same as in Fig. 4.

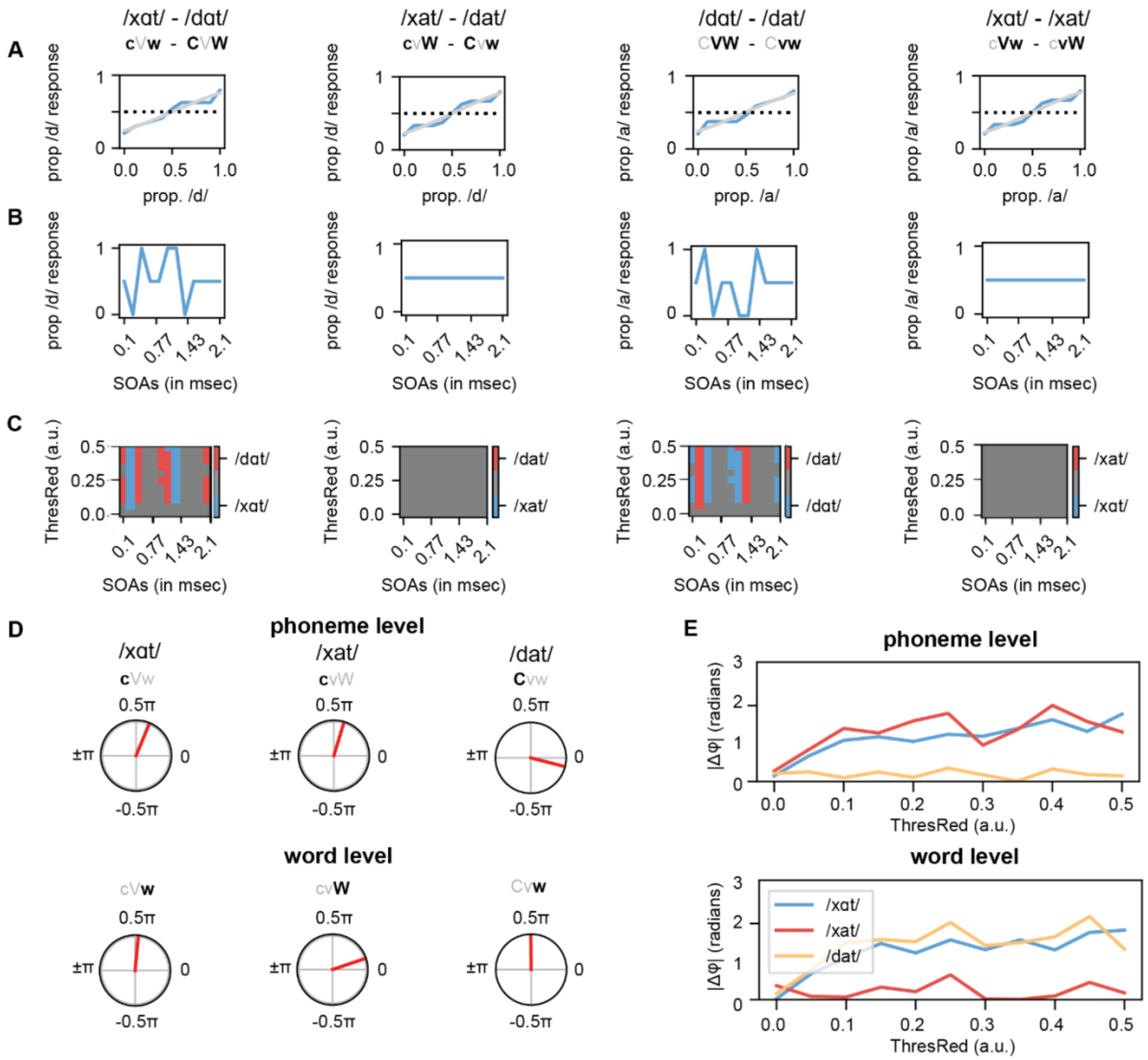

Fig. S14. Outcomes of the computational model when using a 1 Hz oscillator. The entrainment frequency is also adjusted, but the duration of the stimuli and activation function remained the same. All conventions are the same as in Fig. 4.

Tables S1 to S2

Table S1

Proportions of the ambiguity chosen for Figure 4

ThresRed	/xɑt/-/dɑt/	/xat/-/dat/	/dɑt/-/dat/	/xɑt/-/xat/
0	0.5	0.5	0.5	0.5
0.05	0.5	0.5	0.5	0.5
0.10	0.5	0.5	0.5	0.5
0.15	0.5	0.5	0.5	0.5
0.20	0.5	0.5	0.5	0.5
0.25	0.4	0.5	0.6	0.5
0.30	0.4	0.5	0.6	0.5
0.35	0.4	0.5	0.6	0.5
0.40	0.4	0.5	0.6	0.5
0.45	0.3	0.5	0.7	0.5
0.50	0.3	0.5	0.7	0.5

Proportion of the stimulus at the upper half of the contrast (e.g., for /xat/-/dat/ the proportion /dat/) per contrast and threshold reduction level corresponding to Fig. 4. ThresRed = threshold reduction.

Table S2

Proportions of the ambiguity chosen for Supplementary Figure 8

ThresRed	/xɑt/-/dɑt/	/xat/-/dat/	/dɑt/-/dat/	/xɑt/-/xat/
0	0.5	0.5	0.5	0.5
0.01	0.5	0.5	0.5	0.5
0.02	0.5	0.5	0.5	0.5
0.03	0.5	0.5	0.5	0.5
0.04	0.5	0.5	0.5	0.5
0.05	0.5	0.5	0.5	0.5
0.06	0.5	0.5	0.5	0.5
0.07	0.5	0.5	0.5	0.5
0.08	0.4	0.5	0.6	0.5
0.09	0.4	0.5	0.6	0.5
0.10	0.4	0.5	0.6	0.5

Proportion of the stimulus at the upper half of the contrast (e.g., for /xat/-/dat/ the proportion /dat/) per contrast and threshold reduction level corresponding to SI Appendix Fig. 8. ThresRed = threshold reduction.

SI References

1. Praat: a system for doing phonectics by computer v. 5.3.56 (2013).
2. ten Oever, S. & Sack, A. T. Oscillatory phase shapes syllable perception. *Proc Natl Acad Sci U S A* **112**, 15833-15837 (2015). https://doi.org:10.1073/pnas.1517519112
3. Moulines, E. & Charpentier, F. Pitch-synchronous waveform processing techniques for text-to-speech synthesis using diphones. *Speech communication* **9**, 453-467 (1990).
4. Andersen, N. On the calculation of filter coefficients for maximum entropy spectral analysis. *Geophysics* **39**, 69-72 (1974).
5. Brainard, D. H. The Psychophysics Toolbox. *Spat Vis* **10**, 433-436 (1997).
6. Westner, B. U. *et al.* A unified view on beamformers for M/EEG source reconstruction. *NeuroImage* **246**, 118789 (2022). https://doi.org:10.1016/j.neuroimage.2021.118789
7. Cohen, M. X. *Analyzing neural time series data: theory and practice*. (MIT press, 2014).
8. Maris, E. & Oostenveld, R. Nonparametric statistical testing of EEG- and MEG-data. *J. Neurosci. Methods* **164**, 177-190 (2007). https://doi.org:10.1016/j.jneumeth.2007.03.024
9. Zar, J. H. *Biostatistical Analysis*. 4 edn, (Prentice Hall, 1998).
10. Fisher, N. I. *Statistical analysis of circular data*. (Cambridge University Press, 1995).
11. Ten Oever, S. & Martin, A. E. An oscillating computational model can track pseudo-rhythmic speech by using linguistic predictions. *Elife* **10**, e68066 (2021). https://doi.org:10.7554/eLife.68066
